# Stacked convolutional neural network for emotion recognition using multi feature speech analysis

**DOI:** 10.1038/s41598-025-28766-0

**Published:** 2025-11-26

**Authors:** Chandradip Roy, Wreet Kharel, Pijush Das, Ranjit Panigrahi, K. S. Hareesha, Moumita Pramanik, Akash Kumar Bhoi

**Affiliations:** 1 Department of Computer Application, Sikkim Manipal Institute Technology, Sikkim Manipal University, Gangtok, India; 2 Department of Artificial Intelligence, Amrita School of Artificial Intelligence, Amrita Vishwa Vidyapeetham, Faridabad, 121002 India; 3 Department of Data Science & Computer Applications, Manipal Institute of Technology, Manipal Academy of Higher Education, Manipal, 576104 Karnataka India; 4 Department of Electronics and Communication Engineering, Graphic Era Deemed to be University, Dehradun, 248002 Uttarakhand India

**Keywords:** SCoNN, RAVDESS, TESS, SAVEE, Emotion recognition, Multi feature speech analysis, Health care, Psychology

## Abstract

Remote diagnosis is increasingly incorporating emotion recognition, enabling clinicians to assess patients’ emotional states during teleconsultations through analysis of vocal and acoustic characteristics. This study proposes a refined deep learning framework for emotion recognition from speech signals, designed to enhance the reliability of remote medical assessments. Several deep learning architectures, including convolutional neural networks (CNN), recurrent neural networks (RNN), and long short-term memory (LSTM) models, were evaluated using three publicly available emotional speech datasets: RAVDESS, TESS, and SAVEE. The primary contribution of this work is the Stacked Convolutional Network (SCoNN), a deep neural architecture developed to hierarchically extract and integrate complex audio features for improved emotion classification. The model comprises multiple Conv1D blocks incorporating batch normalization, dropout, and activation layers, followed by a dense softmax output layer for final classification. SCoNN achieved accuracies of 99.93% on the TESS dataset using combined MFCC and Mel Spectrogram features; 91.51%, 90.63%, and 93.30% on the RAVDESS dataset for Mel Spectrogram, MFCC, and combined features, respectively; and 91.43%, 94.76%, and 95.00% on the SAVEE dataset for the same feature configurations. The novelty of SCoNN lies in its hierarchical stacking mechanism and adaptive multi-feature fusion, enabling superior capture of emotional variations in speech compared to conventional deep CNNs. The proposed framework demonstrates high efficiency and reliability for emotion recognition in remote healthcare applications.

## Introduction

Humans are inherently emotional beings, and the main attribute that contributes to their social nature is emotion. Emotion is of paramount importance in an individual’s daily life. It facilitates understanding of one another’s internal thoughts and emotions, even without spoken communication. In a hypothetical scenario, a speaker’s smile can symbolize a pleasant sentiment throughout the conversation, while a frown can symbolize an adverse feeling. Positive emotions can elicit a sense of friendliness in the observer, whereas negative emotions might lead to the observer disregarding the speaker. In addition, emotions exert several influences on our daily lives. These influences may have both positive and negative effects. Negative emotions can lead to several severe ailments. These not only induce mental disorders but also give rise to severe bodily ailments. Extreme emotional imbalance has the potential to give rise to a range of ailments. Multiple studies have demonstrated that emotional imbalance has the potential to induce the development of conditions such as bipolar disorder^1,2^, posttraumatic stress disorder (PTSD)^3^, and cardiovascular disease^4,5^. Properly identifying human emotions early can resolve the aforementioned concerns, enabling appropriate measures to be implemented early. In addition to its medical applications, emotion recognition has the potential to enhance human‒machine interactions, enabling the system to offer users a more personalized and convenient experience. Furthermore, the utilization of emotion detection techniques can also help researchers gain a greater understanding of human emotions and behavior more efficiently.

Human emotions can be identified using various biomarkers^6,7^ that reflect an individual’s affective state. Facial expressions are widely used indicators where muscle movements reveal emotions such as happiness or anger. However, facial analysis is limited to face-to-face interactions and is unsuitable for remote applications where visual cues are unavailable. In such cases, voice signals^8^ serve as reliable biomarkers for emotion recognition. Variations in tone, pitch, intensity, and speaking rate occur naturally with changes in emotional state, providing measurable acoustic patterns that can be analyzed computationally.

Researchers have explored several acoustic features—such as pitch, amplitude, intensity, formants, and jitter—to improve emotion detection from speech. Machine learning methods, including K-nearest neighbors (KNN)^9^, support vector machines (SVM)^10^, and decision trees^11^, have been used to classify emotions based on these features. With recent advances in deep learning, models such as recurrent neural networks (RNNs)^12^, convolutional neural networks (CNNs)^13^, artificial neural networks (ANNs)^14^, and long short-term memory (LSTM) networks^15^ have demonstrated superior capability in capturing intricate temporal and spectral patterns in speech. RNNs and LSTMs effectively model sequential dependencies, while CNNs identify local patterns in spectrograms. The integration of diverse vocal parameters within these architectures enhances sensitivity to subtle emotional variations, enabling the development of robust and accurate emotion recognition systems.

This study presents an advanced method to improve emotion recognition during remote medical consultations, focusing specifically on analyzing voice signals as robust indicators of emotional states. Initially, various deep learning algorithms, including convolutional neural networks (CNNs), recurrent neural networks (RNNs), and long short-term memory (LSTM) networks, were explored. These algorithms are applied to publicly available emotion speech datasets such as RAVDESS, TESS, and SAVEE, creating a solid foundation for the efficient and accurate training of models. A significant contribution of this study is the introduction of a sophisticated layered deep learning approach known as the stacked convolutional network (SCoNN). This neural network architecture is carefully designed to enhance the efficiency of emotion recognition by hierarchically extracting complex audio features from voice signals. The SCoNN architecture comprises multiple layers of Conv1D convolutional blocks, each incorporating essential components such as batch normalization, dropout, and activation functions. These layers combine extracting and process audio features hierarchically, resulting in more accurate emotion recognition. The architecture culminates in a dense output layer that utilizes softmax activation, enabling effective discrimination among different emotional states.

The contributions of the article are as follows:

- The exploration and application of deep learning algorithms such as CNNs, RNNs, and long short-term memory (LSTM) networks on publicly available emotion speech datasets such as RAVDESS, TESS, and SAVEE provide a robust training environment for emotion recognition models.
- The stacked convolutional network (SCoNN) architecture, a sophisticated deep neural network designed for efficient emotion recognition by hierarchically extracting complex audio features from voice signals, was introduced.
- The essential components in the SCoNN architecture, including Conv1D convolutional blocks, batch normalization, dropout, activation functions, and a dense output layer with softmax activation, are utilized to ensure effective discrimination among different emotional states.

## Literature survey

Many authors have proposed various methods for emotion detection using machine learning and deep learning approaches. Emotion recognition has become a significant area of research due to its wide-ranging applications in fields such as healthcare, human-computer interaction, and affective computing. Machine learning algorithms such as support vector machines (SVMs), K-nearest neighbors (KNNs), decision trees, and random forests have been widely explored for their ability to classify emotions based on input features. In recent years, deep learning techniques have gained prominence for their ability to automatically learn complex patterns and representations from raw data. Convolutional neural networks (CNNs), recurrent neural networks (RNNs), long short-term memory (LSTM) networks, and their variants have shown remarkable performance in emotion recognition tasks, especially when dealing with complex datasets such as audio signals or facial expressions.

Issa et al.^16^ proposed a 1-D CNN using multiple audio features—chromagram, MFCC, tonnetz, Mel-scale spectrogram, and spectral contrast—across RAVDESS, Emo-DB, and IEMOCAP datasets. While their results were promising, the approach relied heavily on dataset-specific tuning, and generalization across datasets was not fully explored. This indicates a need for models that can retain accuracy across diverse emotional corpora without extensive reconfiguration. Nivedita et al.^17^ examined the use of autoencoders for dimensionality reduction and demonstrated their advantage over PCA in improving recognition accuracy on RAVDESS and TESS datasets. However, the study was limited to basic autoencoders and traditional classifiers such as SVM and decision trees. Replacing these with convolutional or recurrent networks could have captured richer temporal–spectral dependencies in emotional speech.

Similarly, an adaptive frequency coefficient–based feature extraction method was proposed in^17^ which fused fractional Fourier transform and cepstral features using an SVM with an RBF kernel. Despite reporting strong performance on EMO-DB, SAVEE, and PDREC datasets, the work relied on conventional classifiers. Integrating such hybrid features into a deep learning framework could potentially improve robustness and cross-corpus adaptability. Lalith et al.^18^ developed a DNN with three hidden layers to classify seven emotions using the Berlin speech emotion corpus and various feature extraction techniques, including MFCC, LPC, and Fourier parameters. Although the study compared a broad set of features, it did not explore architectural optimization, leaving scope for models that can hierarchically integrate multi-feature representations. Marta et al.^19^ investigated Mel spectrograms and standard spectrograms using ResNet18 and a custom CNN, highlighting the risks of poor data partitioning and its impact on model reliability. Despite using five major datasets, their cross-corpus performance remained modest (57.42%), emphasizing the challenge of building generalizable models without data augmentation or hierarchical feature fusion. Alnuaim et al.^20^ MLP and CNN models with MFCC, STFT, and Mel spectrogram features on RAVDESS. While the MLP achieved 81% accuracy, the work did not address class imbalance, which can bias model evaluation. Addressing this limitation could enhance reliability in multiclass emotion recognition. A hybrid LSTM–Transformer model was proposed in^21^ to capture long-term dependencies using MFCC-based representations. Although the framework achieved high accuracy, it was computationally intensive and required dataset-specific tuning. This underscores the importance of architectures that balance performance and efficiency. Finally, Kumaran et al.^22^ combined MFCC and GFCC features (M-GFCC) with a deep convolutional recurrent neural network, improving recognition performance beyond 80%. However, the framework lacked explicit hierarchical organization of convolutional blocks, which could further enhance discriminative feature learning.

Kakuba et al.^23^ have proposed an attention-based multilearning (ABMD) model. The proposed model uses residual dilated causal convolution blocks (RDCCs) and DC or dilated convolution layers with multihead attention. This study used three publicly available datasets: SAVEE, RAVDESS, and Emo-DB. The spectral and voice quality features, such as the MFCC, Mel spectrogram, and chromagram, are extracted from the voice signals used for training the proposed model. The proposed model achieved 93.75%, 85.89%, and 95.93% accuracy on the SAVEE, RAVDESS, and Emo-DB datasets, respectively. An artificial neural network (ANN) was introduced to improve human-computer interactions^18^. This study used various publicly available speech emotion datasets, such as TESS, RAVDESS, CREMA-D, and SAVEE. The proposed ANN model was trained on MFCC features extracted from each audio file in the dataset. The model achieved accuracies of 88.72%, 71.96%, 99.52%, and 86.80% on the RAVDESS, CREMA-D, TESS, and SAVEE datasets, respectively. Three data augmentation techniques were applied^24^ to improve the robustness of a CNN model for speech emotion detection. The study was conducted on three publicly available datasets: RAVDESS, SAVEE, and Emo-DB. Seven different features are extracted from the voice signals in the datasets, such as spectral contrast, tonnetz, MFCCS, delta-MFCCS, delta-delta MFCCS, and chromagram. The extracted feature vector was fed to the proposed CNN model and achieved accuracies of 96.7% (with seven classes), 90.6% (with 8 classes), and 93.2% (with 7 classes) on Emo-DB, RAVDESS, and SAVEE, respectively. For the cross-corpus with 1930 utterances, the proposed framework achieved 93.3% accuracy in six emotion classes. Tellai et al.^25^ proposed a dual-stream CNN-transformer fusion model for detecting emotion from voice signals. They extracted MFCCs and mel spectrograms from voice signals from three datasets: RAVDESS, TESS, and Emo-DB. In this study, they used two of the abovementioned architectures, the first of which used MFCC and the second of which used a Mel Spectrogram. The output from the architecture is then fused based on the probabilities given by the “SoftMax” activation function.

Recent research has explored transformer-based and attention-driven models for emotion recognition, which capture long-range temporal dependencies effectively. However, such models often demand large datasets and high computational resources, limiting their use in practical applications. The proposed Stacked Convolutional Network (SCoNN) addresses this by using a hierarchical convolutional stacking strategy that extracts temporal–spectral features at multiple resolutions with lower complexity. In related domains, Kumar et al.^26^ proposed an efficient deep learning framework for brain cancer identification, and Yadav et al.^27^ analyzed signal processing methods for mental state recognition in Brain–Computer Interfaces. Both studies highlight the role of effective feature extraction in modeling human affective or physiological signals. Building on these insights, SCoNN combines multi-scale feature learning with efficient convolutional representation to bridge the gap between conventional CNN–LSTM models and recent transformer-based approaches.

Collectively, prior studies demonstrate steady progress in speech emotion recognition, yet many approaches either depend on handcrafted features, lack scalability, or do not integrate multiple feature hierarchies efficiently. These limitations motivate the development of the proposed Stacked Convolutional Network (SCoNN), which aims to achieve higher generalization and feature diversity by hierarchically stacking convolutional layers and leveraging multi-feature fusion for robust cross-dataset emotion detection.

## Materials & methods

This section extensively discusses the datasets utilized, the extraction of different signal features from audio files, and the use of various deep learning methodologies to distinguish between different emotion classes. Furthermore, it outlines the proposed deep learning framework tailored for emotion detection and thoroughly explains the emotion detection process.

### Emotion datasets

For the task of emotion detection, multiple datasets are available, including several publicly accessible ones that contain both video and audio recordings. In this study, we selected three widely used datasets: TESS, RAVDESS, and SAVEE. A brief overview of each dataset is provided below.

#### TESS

**Fig. 1:**
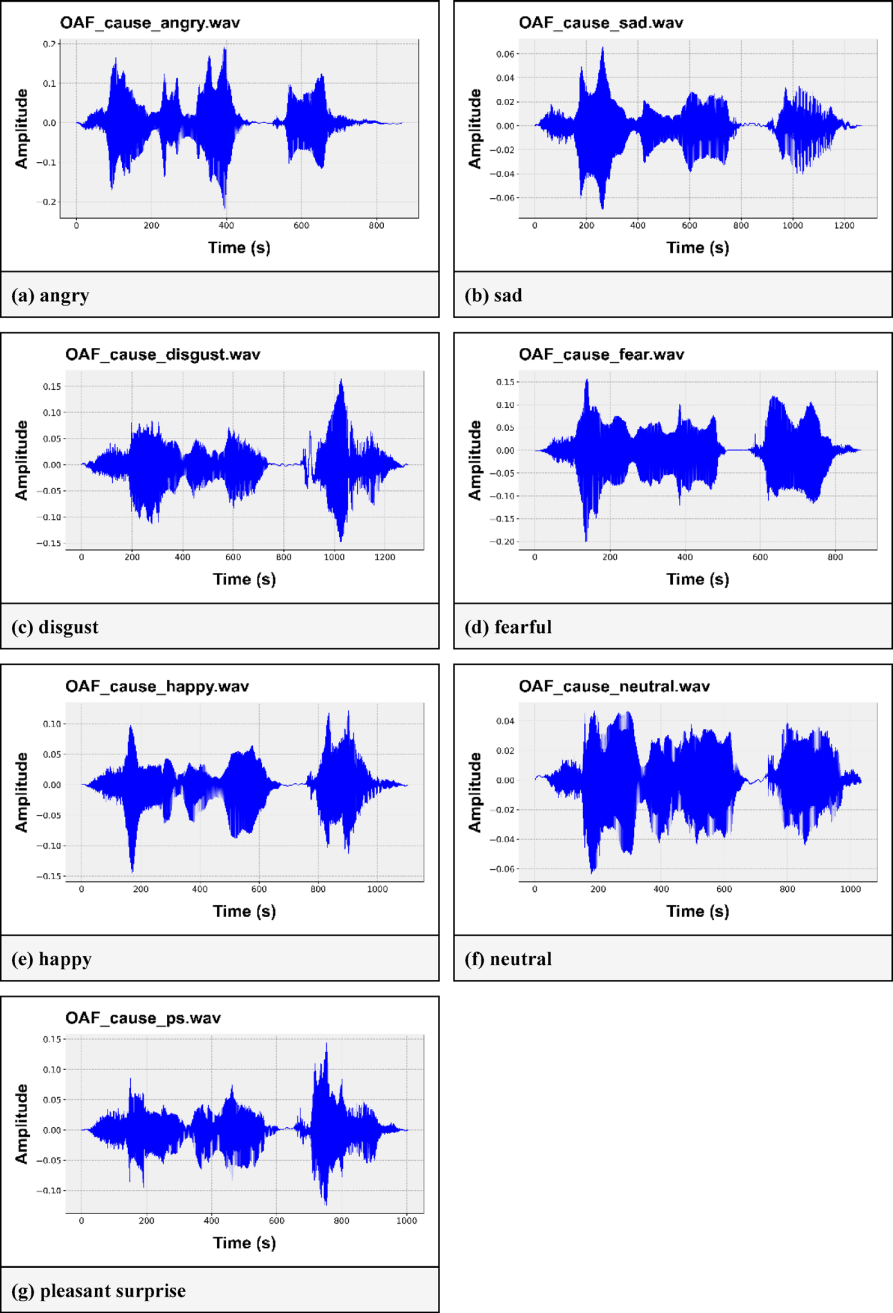
The audio waveforms of different emotion classes in the TESS dataset. (**a**) angry, (**b**) sad, (**c**) disgust, (**d**) fearful, (**e**) happy, (**f**) neutral, (**g**) pleasant surprise.

The Toronto Emotional Speech Set dataset (TESS)^28^ comprises 2800 audio files. Two female voice actresses, aged 26 and 64 years, were recorded speaking 200 target words in the carrier phrase “Say the word____”. These audio files portray seven emotions: neutral, anger, disgust, happy, fearful, pleasant, surprise, and sad. The dataset’s popularity lies in its equal audio samples for each emotion class, with 400 samples for each class. This makes it the most widely used dataset in speech emotion detection. Figure 1 shows the audio waveforms of various emotion labels (angry, disgust, fearful, happy, neutral, sad, and surprised) present in the TESS dataset. Here, it can be seen that each of the emotions has its own wave pattern. This characteristic can help us differentiate among various emotions.

#### RAVDESS

**Fig. 2:**
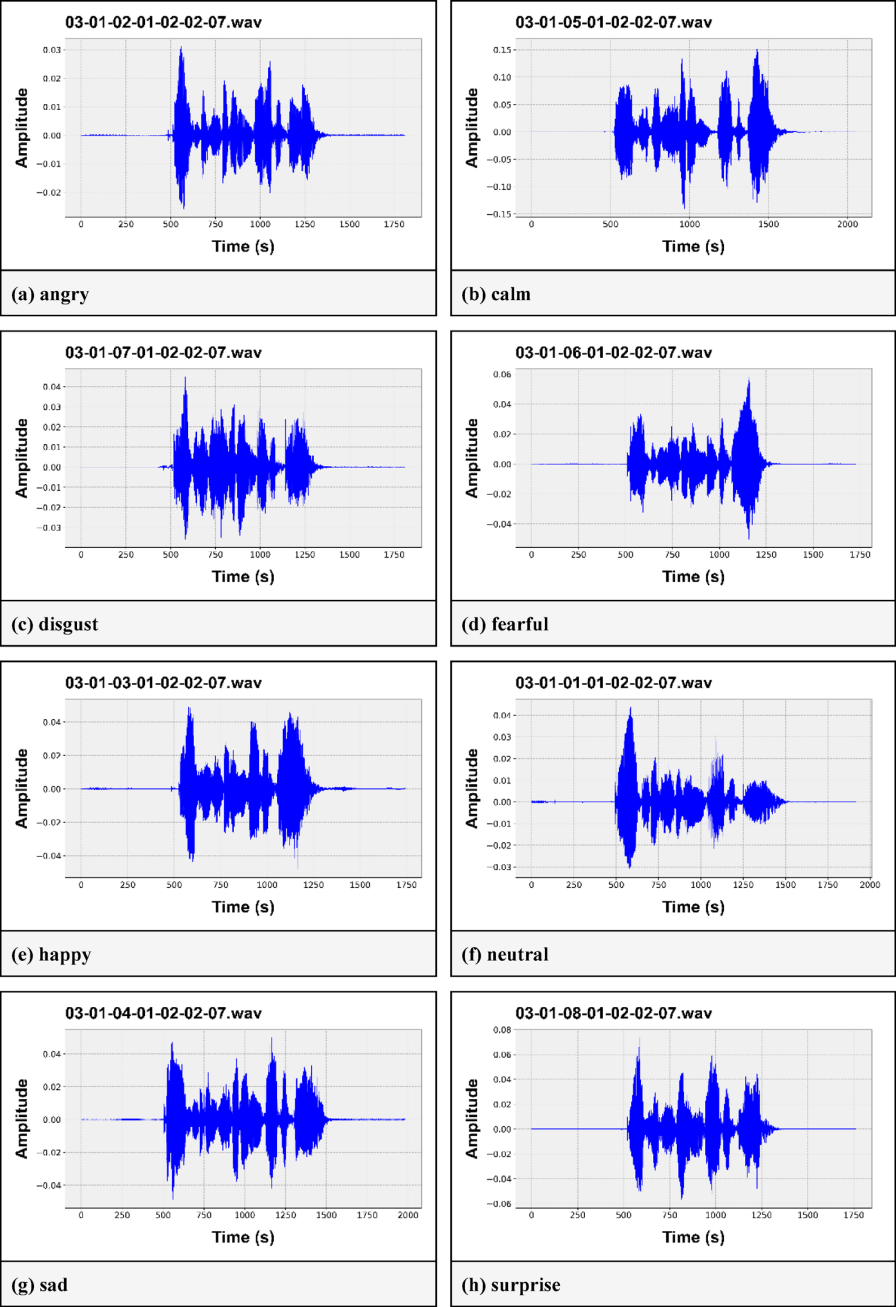
Waveforms of audio of different emotion classes in the RAVDESS dataset. (**a**) angry, (**b**) calm, (**c**) disgust, (**d**) fearful, (**e**) happy, (**f**) neutral, (**g**) sad, (**h**) surprise.

The Ryerson Audio-Visual Database of Emotional Speech and Song (RAVDESS)^29^ dataset is composed of two types of data: speech and song. It comprises audio and video recordings and includes 7356 files recorded by 24 professional actors (12 men and 12 women), with only 1440 being audio speech files. The dataset represents eight different emotions, calm, neutral, happy, angry, sad, disgustful, fearful, and surprised, and each of them is represented by two intensity levels: normal and strong. However, there is an unbalanced distribution of samples for the emotion labels, as there are only 96 samples for the “neutral” class. Figure 2 shows the audio waveforms of various emotion labels present in the RAVDESS (angry, calm, disgust, fearful, happy, neutral, sad, and surprised) dataset. Here, we can see that each of the emotions has its own wave pattern. This characteristic can help us differentiate the various emotions from each other.

#### SAVEE

**Fig. 3:**
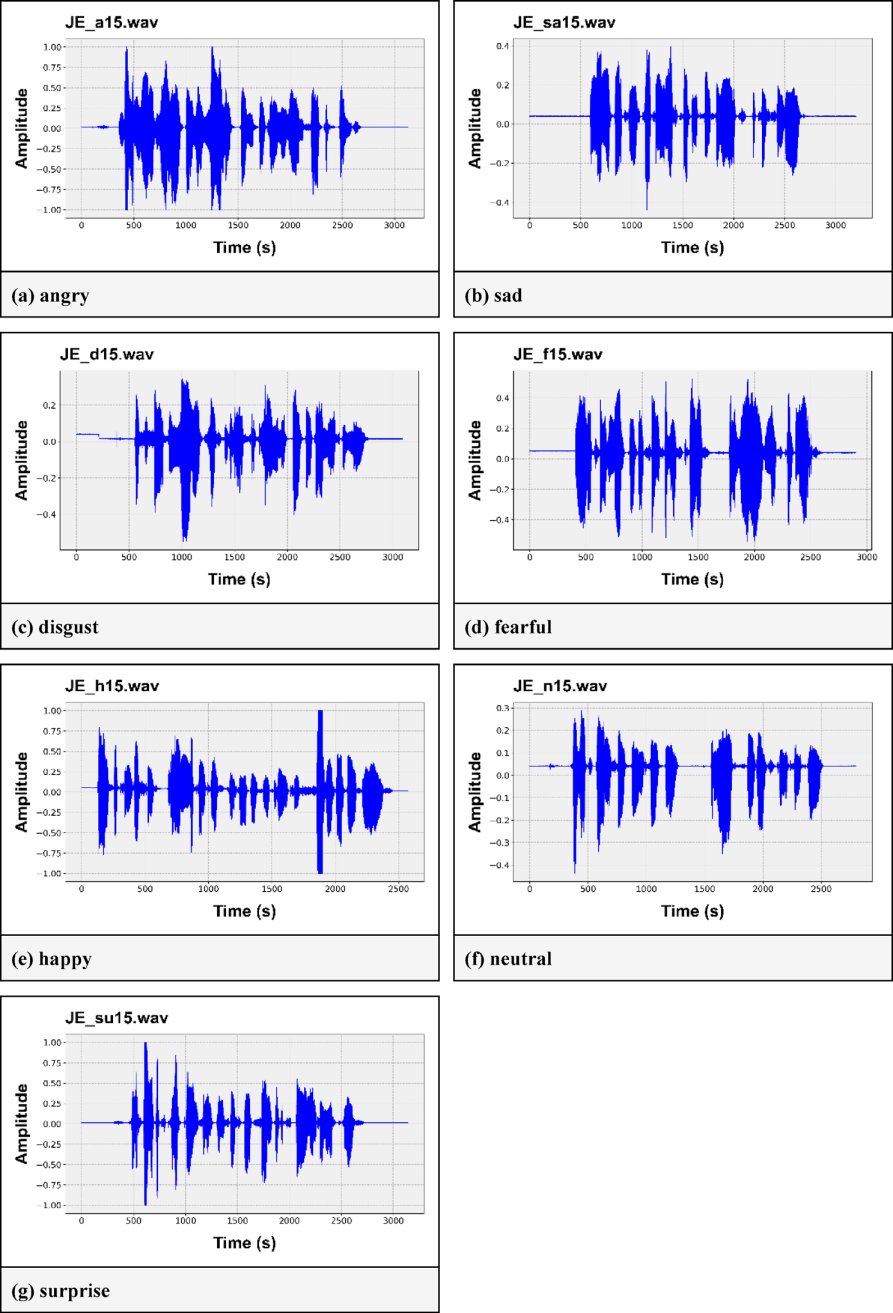
Waveforms of different emotions in the SAVEE dataset. (**a**) angry, (**b**) sad, (**c**) disgust, (**d**) fearful, (**e**) happy, (**f**) neutral, (**g**) surprise.

The SAVEE dataset stands for Surrey Audio-Visual Expressed Emotion, and it includes a total of 480 audio samples^30^These samples were recorded by four English males aged 27 to 31. The dataset comprises seven different emotions: neutral, angry, disgusted, fearful, happy, sad, and surprised. However, the dataset has a class imbalance issue because the ‘neutral’ class has twice as many audio files as the other classes. Figure 3 shows the audio waveforms of various emotion labels present in the SAVEE (angry, disgust, fearful, happy, neutral, sad, and surprised) dataset. Here, we can see that each of the emotions has its own wave pattern. This characteristic can help us differentiate the various emotions from each other.

Table 1 displays the number of samples of different emotions in three datasets—RAVDESS, TESS, and SAVEE. The emotions included calm, neutral, happy, angry, sad, disgusted, fearful, and surprised. In the RAVDESS dataset, there are 192 samples for each of the Calm, Happy, Angry, Sad, Disgust, Fearful, and Surprised emotions, while 96 samples belong to the Neutral emotion. This dataset has a total of 1440 samples. The TESS dataset consists of 400 samples for each emotion class, except for Calm, which has no samples, resulting in 2800 samples. The SAVEE dataset has no samples for calm, 120 samples for neutral, and 60 samples for each of the happy, angry, sad, disgusted, fearful, and surprised emotions. This dataset has a total of 480 samples. This table clearly summarizes the data distribution among various emotional states across these three datasets. It is useful for understanding the dataset’s composition and identifying potential biases in emotion representation.

**Table 1:** The total number of samples in the dataset and the total number of samples available for each emotion class.

Datasets	Calm	Neutral	Happy	Angry	Sad	Disgust	Fearful	Surprised	Total
RAVDESS	192	96	192	192	192	192	192	192	1440
TESS	-	400	400	400	400	400	400	400	2800
SAVEE	-	120	60	60	60	60	60	60	480

### Methodology

**Fig. 4:**
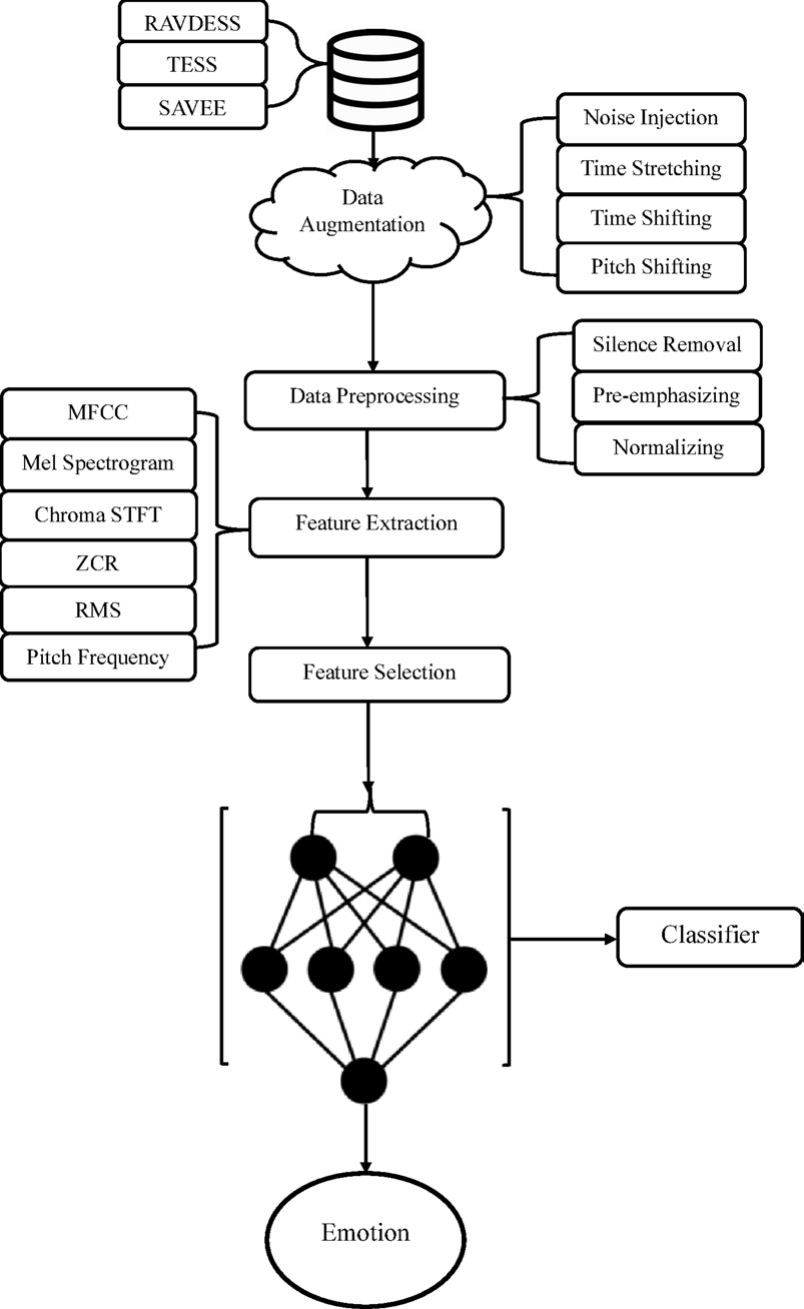
The proposed solution workflow using a stacked convolutional network (SCoNN).

A new deep learning-based model called the stacked convolutional network (SCoNN) has been proposed to address the problem of emotion classification from voice signals. The model utilizes CNNs, RNNs, or long short-term memory (LSTM) networks due to their ability to handle the complexity of voice signals. The proposed SCoNN deep neural network architecture is designed to enhance emotion recognition efficiency by hierarchically extracting complex audio features from voice signals. Additionally, convolutional autoencoders have been used to reduce the dimensionality of extracted features from acoustic signals, as outlined in the literature reviews. To train and validate the model, three publicly available emotional datasets—RAVDESS, TESS, and SAVEE—have been used, along with synthetic data generated from the original audio using four data augmentation techniques: noise injection, time stretching, time shifting, and pitch shifting. Before the audio files are fed into the proposed deep learning model, various preprocessing techniques, including preemphasis, framing, and windowing, are used to normalize and denoise the signals. Based on prior studies, feature extraction involves capturing crucial features for emotion detection, such as MFCCs, LPCCs, the zero-crossing rate, formants, pitch, intensity, and spectrograms. After model training with selected features, real-world data were used to evaluate the model’s performance. Figure 4 is a visual representation of the proposed solution workflow.

The methodology starts with loading the audio files from each dataset for preprocessing. The preprocessing tasks involve the following:

#### Data augmentation

Training a neural network usually requires a large number of samples to achieve good accuracy and generalizability. However, the dataset mentioned in this context has a low number of samples for each class, which can lead to low model accuracy and poor generalizability to unseen data. Moreover, the recordings in the dataset are from a controlled environment with minimal noise, whereas real-world data often contain various types of noise. Four data augmentation techniques are used to address these issues and improve the model’s performance and generalizability. These techniques include noise injections, time stretching, time and pitch shifting. The primary objective of these techniques is to increase the effective size of the dataset by creating variations in the existing samples.

In the context of data augmentation, three example audio files, namely, DC_h15.wav, YAF_doll_happy.wav, and 03-01-01-01-01-01-19.wav, of the SAVEE, TESS, and RAVDESS datasets, respectively, are utilized to demonstrate the pre- and post-augmentation phases across four stages, *namely*, noise injection, time stretching, time shifting, and pitch shifting.

#### Noise injection

The noise injection method is a data augmentation technique used to create variations in datasets by intentionally adding noise to the original signal. This is done by using a function that takes the original waveform of the signal as input and adds random values to it. The random values are generated from a normal distribution with a rate of 0.035, indicating the level of noise to be added. By adding noise to the signal, we simulate real-world conditions where environmental or recording artifacts often affect signals. This technique helps the neural network learn to be robust to noise and enhances its ability to generalize to unseen data with varying levels of disturbance.

For the original signal waveform $\:x\left(t\right)$ over time $\:t$, the signal after adding noise is:

1 $$\:{x}_{noisy}\left(t\right)=x\left(t\right)+ \epsilon \left(t\right)$$

Here, $\epsilon\left(t\right)\:$ is the noise component at time $\:t$. The noise component $\epsilon\left(t\right)$ is estimated from a normal distribution with mean $\:0$ and a specified standard deviation $\:\sigma\:$, denoted as $\:\mathcal{N}\left(0,{\sigma\:}^{2}\right)$. In our case, where noise is added from a normal distribution at a rate of 0.035, the standard deviation $\:\sigma\:$ can be calculated as:

2 $$\:\sigma\:=0.035\times\:\mathrm{m}\mathrm{a}\mathrm{x}\mathrm{i}\mathrm{m}\mathrm{u}\mathrm{m}\:\mathrm{a}\mathrm{m}\mathrm{p}\mathrm{l}\mathrm{i}\mathrm{t}\mathrm{u}\mathrm{d}\mathrm{e}\:\mathrm{o}\mathrm{f}\:x\left(t\right)$$

### Time stretching

This method introduces a technique for altering the duration of a signal along the time axis, either stretching or compressing it^31^. The process involves taking the original waveform and applying a stretching rate, which defaults to 0.8 if not specified. The time stretching operation was performed using the “time_stretch” function provided by the Librosa library.

Time stretching in audio processing typically involves modifying the time axis of a signal while preserving its pitch through a technique called a phase vocoder.

The process of time stretching an audio signal involves several steps. First, the input audio signal is divided into short overlapping frames. Each frame is then transformed into the frequency domain using the Fourier transform. This results in a series of spectra that represent the signal over time. Second, the phase information from the short-time Fourier transform (STFT) is extracted for each frame. This phase information is essential for preserving the spectral characteristics of the signal during time stretching. Next, a time stretching factor is applied to adjust the duration of each frame. This factor determines how much each frame should be stretched or compressed in time. Subsequently, the phase information is adjusted based on the time stretching factor. This adjustment ensures that the signal’s spectral components align correctly after stretching. This, in turn, prevents any artifacts such as phase discontinuities or smearing. Finally, the modified spectra with adjusted phases are transformed back to the time domain using the inverse short-time Fourier transform (ISTFT). This step reconstructs the time-stretched audio signal.

### Time shifting

This audio processing method involves time shifting by deliberately displacing a waveform by a random amount, which adds a dynamic element to the audio signal. The process starts by generating a random value from a uniform distribution ranging from − 4 to 4, inclusive. This range is chosen to provide a significant but not extreme shift, which maintains the coherence of the audio while introducing noticeable variation. The generated random value is scaled by 1000 to convert it into milliseconds; a common unit used in audio processing to represent time durations. This conversion ensures that the shift is in a perceptually meaningful range, aligning with typical audio manipulation scenarios. After obtaining the scaled random value, it is rounded to the nearest integer, which facilitates precise time adjustments. This step is crucial for maintaining synchronization with other audio elements or tracks in a composition. The direction of the shift is determined by the sign of the random number: if positive, the waveform is shifted to the right, effectively delaying its onset in time; conversely, if negative, the waveform is shifted to the left, bringing its start closer to the beginning of the timeline.

The concept of time shifting is particularly useful in audio production and sound design. It can be employed to create various effects such as phasing, flanging, and echo, adding depth and richness to the audio. In music production, time shifting is often used to align tracks, create rhythmic variations, or introduce subtle nuances that enhance the overall listening experience. Additionally, in sound processing applications such as speech recognition or audio analysis, time shifting can be utilized for data augmentation. This approach improves the robustness and diversity of training datasets by introducing temporal variations in the input signals.

### Pitch shifting

Pitch shifting is an audio processing technique that changes the perceived pitch of a waveform without significantly affecting its duration or timing. This technique uses the “pitch_shift” function in the Librosa library to introduce a pitch-shift effect to the original waveform. It accepts three parameters: the waveform, the audio sampling rate, and the “pitch_factor” parameter. The “pitch_factor” parameter determines the degree of pitch shift applied to the waveform. Its default value is 0.7, but you can specify a different value. A pitch factor of 0.7 means that a downward pitch shift is applied to the audio, resulting in a lower perceived pitch than that of the original waveform. Conversely, a pitch factor greater than 1.0 produces an upward pitch shift, increasing the perceived pitch. This method allows you to fine-tune the pitch modification to achieve the desired audio effect, such as by creating harmonies, adjusting vocal tones or experimenting with musical arrangements.

The “pitch_shift” function in Librosa uses advanced signal processing techniques to modify the pitch while preserving the audio’s temporal characteristics. This ensures that the pitch-shifted waveform maintains its natural rhythm and timing, making it suitable for various applications, such as audio synthesis, music production, and sound design. By providing control over the pitch factor, this method allows users to creatively manipulate the tonal qualities of audio signals, enhancing the overall richness and expressiveness of the audio content.

Table 2 represents the total number of audio samples after applying the augmentation techniques (including the original samples). Notably, the ‘neutral’ class of the RAVDESS dataset is removed before the next step starts because of the class imbalance issue. The exclusion of the “neutral” class from the RAVDESS dataset was intentional to maintain balanced class distribution across training and testing samples. The number of “neutral” samples in RAVDESS (96) was significantly lower than other emotion categories (192 each), and initial trials showed that this imbalance caused model bias toward majority classes, reducing recognition accuracy for minority emotions. To ensure fair training, this class was omitted, aligning with common practice in emotion recognition studies where severely underrepresented classes are excluded to prevent skewed learning outcomes.

**Fig. 5:**
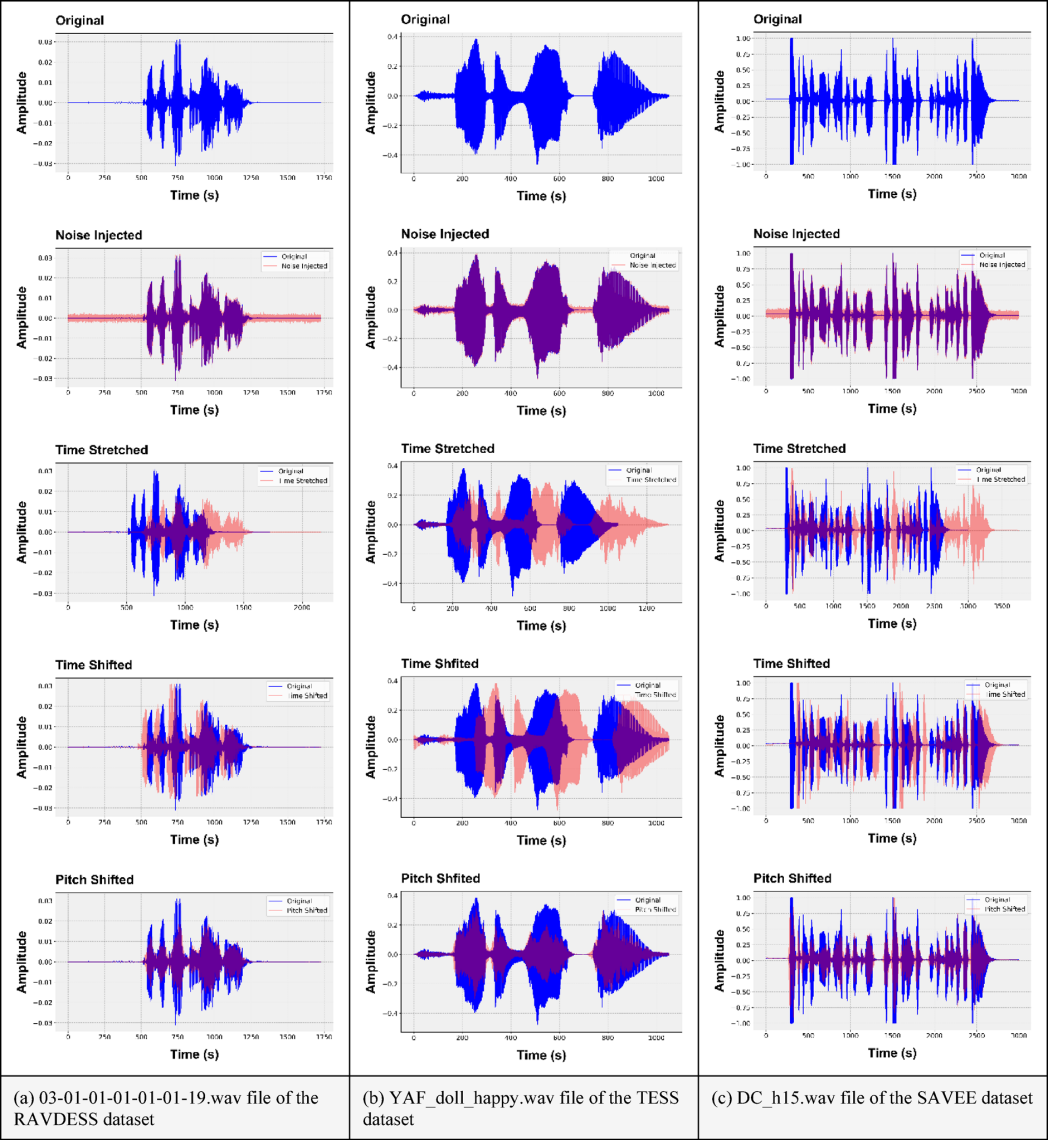
Changes in vocal signals for the RAVDESS, TESS, and SAVEE datasets.

Figure 5 shows the changes in vocal signals through noise injection, time stretching, time shifting, and pitch shifting of a random file from the RAVDESS, TESS, and SAVEE datasets.

**Table 2:** Total audio samples after applying the augmentation techniques (including the original samples).

Datasets	Calm	Neutral	Happy	Angry	Sad	Disgust	Fearful	Surprised	Total
RAVDESS	960	-	960	960	960	960	960	960	6720
TESS	-	2000	2000	2000	2000	2000	2000	2000	14,000
SAVEE	-	300	300	300	300	300	300	300	2100

#### Silence removal

Addressing the unvoiced or silent portions of the signal is important to ensure consistent and accurate results from voice signal processing models. These segments can negatively impact performance and introduce inconsistencies. One approach to address this issue is utilizing an ‘envelope’ method, which eliminates unvoiced segments from the signal based on a specified threshold. These portions can significantly impact the performance and consistency of voice signal processing models, particularly in tasks such as emotion classification. The method involves creating a binary mask, denoted as $\:Mask\left[t\right]$, which indicates signal activity (true) or silence (false) at each time index $\:t$.

3 $$\:Mask\left[t\right]=\left\{\begin{array}{c}True\\\:False\:\end{array}\right.\genfrac{}{}{0pt}{}{if\:mean\left(\left|{y}_{t}\right|\right)>threshold}{otherwise}$$

where $\:\left|{y}_{t}\right|$ represents the absolute value of the input signal at the time index $\:t$. This means that the sign of the signal is ignored, and only its magnitude is considered. Similarly, $\:mean\left(\left|{y}_{t}\right|\right)$ calculates the average of the absolute values within a rolling window at the time index $\:t$. A rolling window means that for each time index, a window of data points around that index is considered, and the mean is calculated within that window. The threshold is a value that serves as a criterion. If the mean of the absolute values within the rolling window at the time index $\:t$ is greater than the threshold, the mask is set to $\:True$; otherwise, it is set to $\:False$. In simpler terms, Eq. 4 checks whether the average magnitude of the signal within a specific time window is above a certain threshold. If it is, the mask is set to $\:True$; otherwise, it is set to $\:False$. This masking operation is commonly used in signal processing to filter out or highlight specific signal parts based on their characteristics. In this case, the silence has been removed.

#### Pre-emphasizing

Pre-emphasis is a technique used in audio signal processing to modify the characteristics of the audio signal. The goal is to enhance specific features or improve the signal’s quality for further processing. In this technique, the masked audio signal undergoes pre-emphasis, which modifies the audio signal to boost the higher frequencies of the voice signal relative to lower-frequency components. This adjustment can improve the clarity and intelligibility of speech. Higher frequencies carry essential details such as consonant sounds and sibilants. A first-order high-pass filter typically implements pre-emphasis, which introduces a linear phase shift and amplifies higher frequencies^32^. Therefore, the pre-emphasized signal at the time index $\:n$ can be expressed as

4 $$\:y\left[n\right]=x\left[n\right]-\alpha\:x\left[n-1\right]$$

Here, $\:x\left(n\right)$ represents the input signal at time index $\:n$. Similarly, $\:x(n-1)$ represents the previous signal at time index $\:n-1$, and $\:\propto\:$ is a coefficient that controls the strength of the recursive filtering effect. It varies between 0.9 and 1.

#### Normalization

After pre-emphasizing the signal, it is important to ensure that the volume of the sound is standardized, which is where normalization occurs^32^. Normalization involves adjusting the amplitude levels of the audio signal to a standard or specified range. This process helps ensure consistency and uniformity across different audio segments or recordings, making it easier to compare, analyze, and process them effectively. Overall, normalization is a crucial step in the audio processing pipeline that helps improve audio data quality and usefulness. The process of normalization is used to regulate the amplitude of an audio signal and bring its levels within a specific range. This is accomplished by scaling the amplitude by a factor that is based on the maximum amplitude present in the signal or a predetermined target amplitude level. In simpler terms, normalization adjusts the volume of the audio signal so that it is not too soft (low amplitude) or too loud (high amplitude). This adjustment is essential to maintain a consistent and comfortable listening experience, especially when dealing with different audio sources or varying recording conditions.

### Vocal feature extraction

Using vocal features is critical for detecting mental disorders and emotional states through deep learning. Variations in various vocal features provide valuable insights into mood shifts, anxiety levels, and cognitive issues. Deep learning models trained on these features have shown remarkable success in identifying patterns linked to depression, anxiety disorders, and neurological conditions. Analyzing patterns in vocal features further enhances emotion detection, enabling rapid classification of different emotional states. These features are essential tools for early detection and personalized interventions, significantly improving mental health outcomes. Various prominent vocal features.

The process of feature extraction from audio files involves identifying and extracting important attributes or characteristics from the audio data. These attributes capture crucial information that can be used for tasks such as analyzing, classifying, or processing audio signals. This process is essential for converting raw audio data into structured and informative representations that can be effectively used in various applications and algorithms related to audio signal processing.

After preprocessing the signal, various techniques are used to extract the essential features. This includes –.

#### Jitter

Small variations in the timing of vocal cord vibrations, referred to as jitter, can indicate instability or uneven vibration in the vocal cords. Jitter can be detected through the following equation, which calculates the average absolute value of jitter and is particularly useful for emotion detection using deep learning:

5 $$\:Jitter=\frac{1}{N-1}\sum\:_{n=1}^{N-1}\left|T\left(n+1\right)-T\left(n\right)\right|$$

Here, $\:T\left(n\right)\:$represents the time of each vibration cycle, and N is the total number of cycles. Calculating jitter using this method provides valuable insights into speech patterns, and emotional cues can be obtained, thus improving the accuracy of deep learning models designed for emotion detection.

#### Shimmer

Shimmer measures slight variations in the loudness of the voice. When there are small ups and downs in how loud a person speaks, shimmer detects that. Similarly to jitter, higher shimmer values can indicate that there might be an issue with the vocal cords, affecting how smoothly the cords vibrate. The following equation is used to calculate the average value of shimmer^33^.

6 $$\:Simmer=\:\frac{\frac{1}{N-1}\sum\:_{i=1}^{N-1}|{A}_{i}-{A}_{i+1}|}{\frac{1}{N}\:\sum\:_{i=1}^{N}{A}_{i}}$$

In addition to identifying vocal cord issues, shimmer is also suitable for emotion detection^34^. Shimmer values increase when a person is experiencing strong emotions such as excitement, anxiety, or stress^35^. Therefore, shimmer analysis can measure emotional intensity in speech. This makes it useful in various fields, including psychology, linguistics, and human-computer interaction.

#### Formants

Formants are frequencies in the acoustic signal produced by the human vocal tract during speech. These frequencies correspond to peaks in the spectrum of the sound wave. When we speak, air from our lungs passes through our vocal cords, creating a fundamental frequency. As this sound travels through our vocal tract, it encounters various obstructions and resonances caused by our tongue, lips, jaw, and other structures. These obstructions and resonances lead to the amplification of certain frequencies, resulting in the formation of formants. There are three different types of formants in our vocal tract, labeled F1, F2, and F3. The first formant (F1) is primarily influenced by the height of the tongue within the oral cavity. A high tongue position corresponds to a lower F1 frequency, while a low tongue position results in a higher F1 frequency. The second formant (F2) is influenced by the front-to-back position of the tongue within the oral cavity. Moving the tongue forward increases the F2 frequency, while moving it backward lowers the F2 frequency. The third formant (F3) is influenced by the rounding or spreading of the lips. Rounded lips lower the F3 frequency, while spread lips increase the F3 frequency.

#### Mel-frequency cepstral coefficient (MFCC)

Mel-frequency cepstral coefficients (MFCCs) are a set of features widely used in speech and audio signal processing, especially in tasks such as speech recognition, speaker identification, and emotion detection. Estimating MFCCs involves several steps, such as signal framing, windowing, Fourier transform calculation, Mel filter bank estimation, logarithmic compression, and discrete cosine transform (DCT) application.

**Step 1. Signal Framing**

Under signal framing, for an audio signal $\:x\left(t\right)$ sampled at a rate of $\:{f}_{s}$ Hz, the continuous-time signal $\:x\left(t\right)$ is divided into frames of duration $\:T$ seconds, overlapping by $\:T/2$ seconds (commonly used values are $\:T=20$ to $\:30$ milliseconds).

**Step 2. Windowing**

Let $\:{x}_{i}\left(n\right)$ represent the $\:i$-th frame of the signal, where is the sample index within the frame. In the windowing step, each frame $\:{x}_{i}\left(n\right)$ is multiplied elementwise with a window function $\:w\left(n\right)$ to reduce spectral leakage, generally using a hamming window:

7 $$\:{x}_{i}\left(n\right).w\left(n\right)$$

**Step 3: Estimating Fourier Transform**

In this step, discrete Fourier transform (DFT) is applied to each windowed frame to convert it from the time domain to the frequency domain:

8 $$\:{x}_{i}\left(k\right)=DFT\left\{{x}_{i}\left(n\right).w\left(n\right)\right\}$$

where $\:{x}_{i}\left(k\right)$ represents the frequency components of the $\:i$-th frame.

**Step 4: Estimating the Mel Filter Bank Energies**

A Mel filter bank with $\:M$ triangular filters was designed, spaced according to the Mel scale. The Mel frequency $\:{f}_{Mel}$ corresponding to a frequency $\:f$ Hz is given by:

9 $$\:{f}_{Mel}=2595.{log}_{10}\left(1+\frac{f}{700}\right)$$

Once the Mel frequency $\:{f}_{Mel}$ is in hand, the Mel filter bank is applied to the magnitude spectrum $\:\left|{x}_{i}\left(k\right)\right|$ to obtain the Mel frequency energies. This is calculated as:

10 $$\:{E}_{i}\left(m\right)=\sum\:_{k=0}^{N-1}{\left|{x}_{i}\left(k\right)\right|}^{2}.{H}_{m}\left(k\right)$$

Here, $\:{E}_{i}\left(m\right)$ is the energy in the $\:{m}_{th}$ Mel filter, $\:{H}_{m}\left(k\right)$ is the response of the $\:{m}_{th}$ Mel filter at frequency bin $\:k$, and $\:N$ is the DFT size.

**Step 5. Logarithmic Compression**

In this step, the dynamic range of $\:{E}_{i}\left(m\right)$ is compressed through logarithmic compression as:

11 $$\:{MFCC}_{i}\left(m\right)=\mathrm{log}\left({E}_{i}\left(m\right)\right)$$

**Step 6. Applying the discrete cosine transform (DCT)**

The discrete cosine transform (DCT) is applied to the log filter bank energies to extract cepstral coefficients.

12 $$\:{C}_{i}\left(p\right)=\sum\:_{m=0}^{M-1}{MFCC}_{i}\left(m\right).\mathrm{cos}\left[\frac{\pi\:p}{M}\left(m+\frac{1}{2}\right)\right]$$

Here, $\:{C}_{i}\left(p\right)$ is the $\:{p}_{th}$ MFCC of the $\:{i}_{th}$frame, and $\:M$ is the number of Mel filters.

#### Chroma STFT

Short-time Fourier transform (STFT) is a method for analyzing the frequency content of a signal over short, overlapping time windows. Given an audio signal $\:x\left(t\right)$, the STFT computes the Fourier transform of the signal within each time window, resulting in a time-frequency representation $\:X\left(t,f\right)$. Chroma short-time Fourier transform (Chroma STFT), on the other hand, is a technique used in audio signal processing to extract chroma-based features from the signal’s frequency content. More precisely, chroma features represent the pitch content of the audio signal, focusing on the relative distribution of musical pitch classes rather than absolute frequency information.

Let $\:{X}_{i}\left(t,f\right)$ be the STFT magnitude spectrum of frame $\:i$ at time $\:t$ and frequency $\:f$. Then, the steps for estimating the Chroma STFT are as follows:

**Step 1. Filter Bank Calculations**

Mathematically, the Chroma filter bank $\:{H}_{i}\left(f\right)$ centered on the pitch class $\:n$ can be defined using triangular-shaped filters. The equation for each filter in the filter bank can be expressed as:

13 $$\:{H}_{n}\left(f\right)=max\left(\mathrm{0,1}-\frac{\left|f-{f}_{n}\right|}{{f}_{bin}/2}\right)$$

Here, $\:f$ is the frequency, $\:{f}_{n}$ is the center frequency of pitch class $\:n$ and $\:{f}_{bin}$ is the frequency resolution of the filter bank, i.e., the distance between adjacent bins in a spectrogram.

**Step 2. Compute Chroma Features**

The chroma feature $\:{C}_{i}\left(n\right)$ frame $\:i$ can be computed as:

14 $$\:{C}_{i}\left(n\right)={\sum\:}_{f}{\left|{X}_{i}\left(t,f\right)\right|}^{2}.{H}_{n}\left(f\right)$$

#### Mel spectrogram

The Mel spectrogram is a combination of the Mel scale and spectrogram. Here, the nonlinear transformation of the frequency scale is represented by the mel scale^36^. The formula below can be used to calculate the Mel spectrogram^37^:

15 $$\:{S}_{mel}\left(k,t\right)=\sum\:_{l=0}^{L-1}{m}_{k}\left(l\right).{\left|S\left(l,t\right)\right|}^{2}$$

Here, $\:{S}_{mel}\left(k,t\right)$ denotes the value of the mel spectrogram at the mel frequency bin $\:k$ and the time frame $\:t$. The magnitude of the spectrogram at the frequency bin $\:l$ and time frame $\:t$ is denoted by $\:S\left(l,t\right)$. Similarly, the value of the mel frequency bin $\:k$ in the frequency bin $\:l$ is denoted by $\:{m}_{k}\left(l\right)$.

#### ZCR (Zero-crossing rate)

The zero-crossing rate (ZCR) is a feature commonly used in audio signal processing to measure the rate at which a signal changes its sign. It provides information about the rapidity of fluctuations in the signal, which can be useful in tasks such as speech recognition, music analysis, and sound classification. The ZCR is calculated as:

16 $$\:{ZCR}_{t}=\frac{1}{2}\:\left(\:\sum\:_{n=1}^{N}\left(sign\left(x\right[n]\right)-sign\left(x\right[n-1])\right)$$

Here, the specimen of the signal at time index $\:n$ is represented by $\:x\left[n\right]$. The $\:sign\left(.\right)$ function returns $\:-1$ for negative values, $\:0$ for zero, and $\:1$ for positive values. The total number of specimens in the signal is represented by $\:N$.

#### Root mean square

The root mean square (RMS) is a statistical measure used in signal processing to calculate the average magnitude of a signal over a specific time period. It is a useful tool for analyzing the energy or power content of a signal. In the context of detecting emotions from audio signals, the RMS can be used as a feature to capture certain aspects related to emotional expression in speech. Specifically, it calculates the overall magnitude of the voice signal. It can be calculated as:

17 $$\:{RMS}_{i}=\sqrt{\frac{1}{N}\sum\:_{n=0}^{N-1}x{\left[n\right]}^{2}}$$

Here, the number of specimens in each frame is denoted by $\:n$. The sample at position n is denoted by $\:x\left[n\right]$.

#### Linear predictive coding (LPC)

Linear predictive coding (LPC) is a technique used in speech and audio processing for modeling the spectral envelope of a signal^38^. It is particularly useful for speech compression, synthesis, and analysis. LPC assumes that a speech signal can be approximated by a linear combination of its past samples.

18 $$\:LPC=\sum\:_{k=1}^{p}{a}_{k}\:x\left(m-k\right)+e\left(m\right)$$

Here, $\:p$ is the order of the LPC analysis, representing the number of coefficients to be estimated. The LPC coefficients to be estimated are represented as $\:{a}_{k}$. The signal samples in the past are presented in $\:x\left(m-k\right)$. The current sample index is $\:m$, and $\:k$ ranges from $\:1$ to $\:p$. The prediction error at time $\:m$ is presented as $\:e\left(m\right)$.

#### Pause frequency

Pause frequency in speech analysis refers to the rate or frequency at which pauses occur in spoken language. Pauses are crucial in speech because they provide cues about the speaker’s thought processes, emphasize certain words or phrases, and help structure the speech into meaningful units. The pause frequency can be estimated as:

19 $$\:PF=\frac{Number\:of\:pauses}{Total\:speech\:duration}\times\:60$$

### Model exploration and analysis

During the execution of this research, several deep learning algorithms, such as CNNs, RNNs, and long short-term memory (LSTM), were investigated. Most studies suggest that these algorithms are state-of-the-art for emotion detection^39^. Rather than evaluating these models in isolation, the study also involved their combination during the evaluation process. Specifically, the research examined a CNN, LSTM, a combination of CNNs with LSTM, and LSTM with RNN and introduced a novel deep CNN architecture termed the stacked convolutional network (SCoNN). Each model, including the newly proposed SCoNN, underwent training with learning rates of 0.00005 and 400 epochs. To mitigate overfitting, early stopping was crucial in the training process.

#### Architecture of the CNN model

A convolutional neural network (CNN) is a type of neural network specifically designed to process and analyze data such as images, audio, and time series. CNNs are composed of one or more convolutional layers. Each convolutional layer uses a set of learnable filters to process the input data. These filters move across the input data, multiply elementwise and sum up the results to generate feature maps. The filters can capture various local patterns and features from the input of data. The feature maps can be calculated as:

20 $$\:Output=Activation\left(\sum\:_{i=1}^{n}\left({W}_{i}\:\times\:{X}_{i}\right)\:+b\right)$$

Here, $\:{W}_{i}$ is the learnable filter, $\:{X}_{i}$ is the input feature map, $\:b$ is the bias term, and $\:Activation\left(\right)$ is the ReLU activation function.

In the context of emotion detection, CNNs have been engineered, focusing on speech signal analysis to detect emotional states. Each sample in the dataset corresponds to a segment of speech, where the input data are structured as (batch_size, number_of_time_steps, 1), denoting the number of time steps in each segment and one feature dimension representing the speech signal.

The CNN model used here consists of a Conv1D layer in multiple stages with a systematic reduction in the filter size, i.e., 256, 128, and 64. The main task of the convolution layer is to process the input speech features to extract relevant local patterns. The convolutional layers are always applied interchangeably with a max pooling layer, which is responsible for gradually reducing the sample size to avoid overfitting. The general structure of the convolutional layer and max pooling layers used for the CNN is:

21 $$Conv1D :=Conv1D (f_{sz}, k_{sz} = 5, striders=1, padding = 'same', activation='relu')$$

22 $$MaxPooling1D := MaxPooling1D (poolsize=5, strider=2, padding='same')$$

**Fig. 6:**
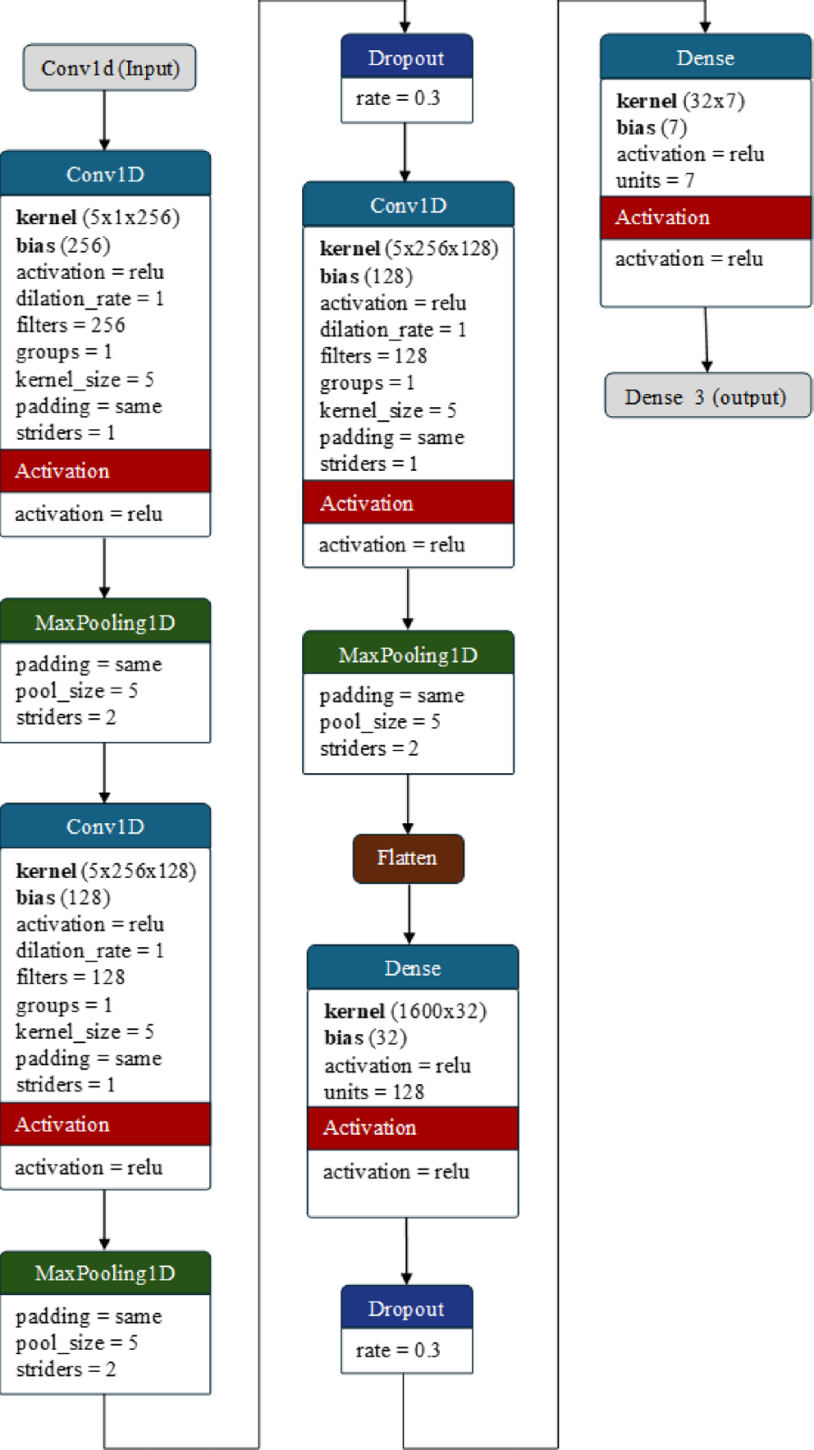
The architecture of the CNN model used for emotion detection.

It should be noted that a traditional deep learning model such as a CNN is highly susceptible to overfitting due to the involvement of a large number of samples. Although the sample size has been significantly reduced by the application of max pooling, it is still sufficient to overfit the neural network. Therefore, to avoid the chances of overfitting, a dropout regularization with a rate of 0.3 is applied to mitigate overfitting by randomly dropping 30% of the units’ outputs during training.

Once the requisite number of feature maps is available, they are reshaped into a vector to be fed into the dense layers. A fully connected dense layer with 32 neurons and a ReLU activation function facilitates the learning of complex representations as follows:

23 $$\:Dense\:Layer:=Dense(units=32,\:activation='relu')$$

Further regularization is carried out through a dropout layer to eliminate any possible overfitting. Finally, the output layer comprises neurons equal to the number of emotion classes in the dataset, employing the softmax activation function to generate class probabilities. The architecture of the CNN model used here is presented in Fig. 6.

#### Architecture of the LSTM model

**Fig. 7:**
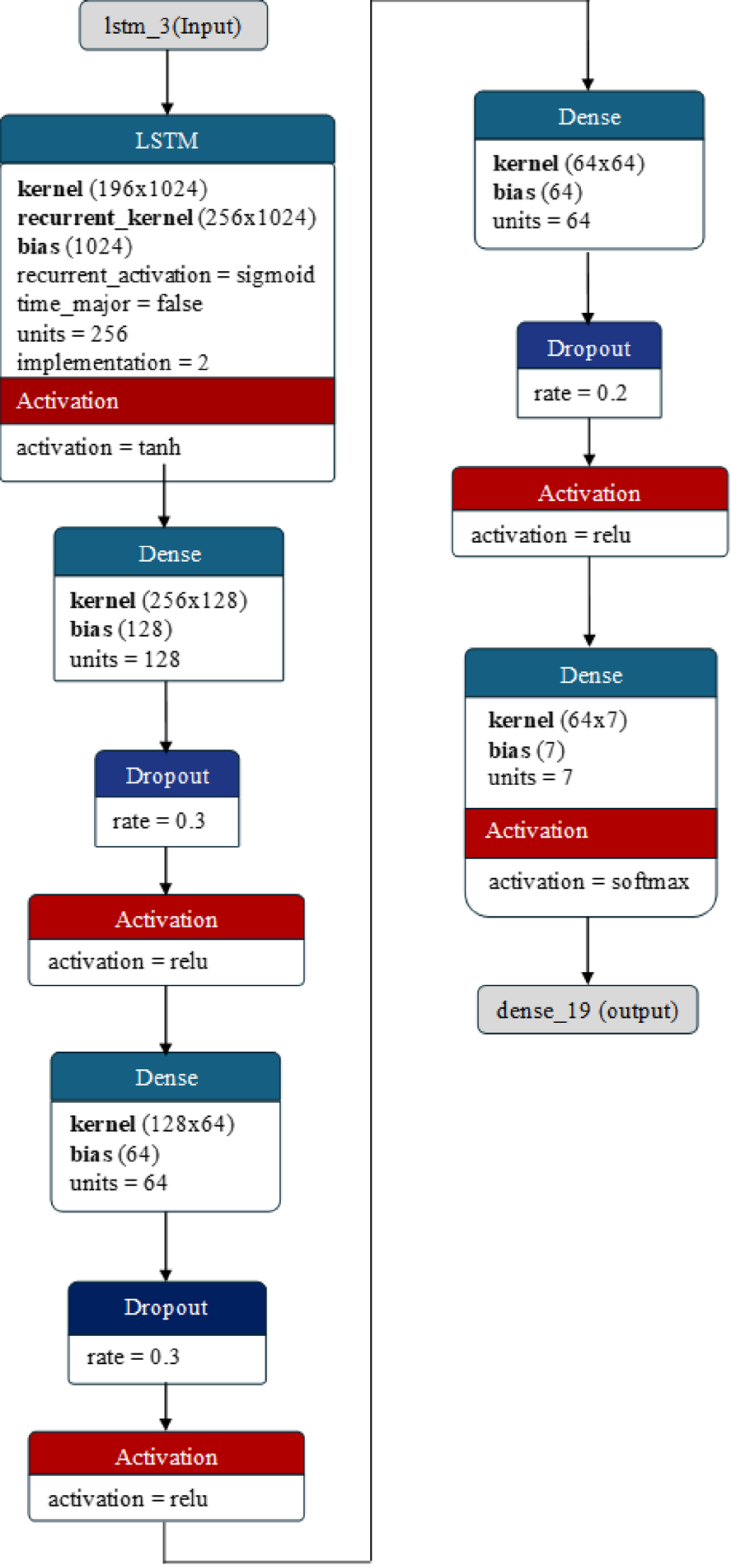
The architecture of the LSTM model used for emotion detection.

LSTM is a type of neural network designed to better understand the dependencies in sequential data by keeping track of a memory cell. This cell can store information over time and selectively update or forget information using a gating mechanism. LSTM was specifically developed to address the vanishing gradient issue that often occurs when training a conventional RNN on long data sequences. LSTMs help to capture temporal dependencies in speech signals. This helps the model learn patterns of emotions expressed over time, aiding in emotion identification. Using sequential features collected from voice signals, such as spectrograms or mel frequency cepstral coefficients (MFCCs), LSTMs can differentiate between variations in vocal characteristics representing different emotional states. These models are proficient at handling sequences of varying lengths, which is essential for analyzing speech segments of different durations. Their ability to learn hierarchical representations enables them to extract relevant features at different levels of abstraction, contributing to accurate emotion recognition. Furthermore, LSTMs can adapt to contextual information within an utterance, providing a deeper understanding of the emotional context in conversations or narratives. Their robustness in handling noisy input ensures reliable emotion detection even in environments with background noise or acoustic distortions, making them suitable for analyzing voice signals in emotion detection tasks. The architecture of the LSTM model used for emotion detection is presented in Fig. 7.

The LSTM layer is the core of the model and is designed to capture long-term dependencies in the input sequences. With 256 units, it has the capacity to learn complex patterns and relationships within the data. $\:return\_sequences=False$This means that the layer outputs only the final time step’s output, often used in sequence-to-vector tasks where the model needs to make a single prediction based on the entire sequence. Following the LSTM layer, a dense layer with 128 neurons and ReLU activation is applied. This dense layer is fully connected to the LSTM layer, allowing it to learn from the rich representations generated by the LSTM. The ReLU activation function introduces nonlinearity, enabling the model to capture more complex patterns in the data. The dense layer can be represented as:

24 $$\:Dense\:Layer\: :=Dense\:(128,\:activation{=}^{\:'}rel{u}^{'}\:)$$

A dropout layer with a 30% dropout rate is added to prevent overfitting after the first dense layer. During training, this layer randomly drops 30% of the units’ outputs, forcing the model to learn more robust and generalizable features. The model includes dense layers with decreasing neuron counts (64 neurons each) and rectified linear unit (ReLU) activation, followed by dropout layers (30% and 20% dropout rates) after each dense layer. These additional layers and dropout mechanisms help the model learn hierarchical representations and avoid overfitting by introducing regularization. Finally, the output layer is a dense layer with the number of neurons equal to the number of unique classes in the target variable (numClasses). The ‘softmax’ activation function is used here, which is ideal for multiclass classification tasks. SoftMax normalizes the output probabilities across classes, making it suitable for predicting class probabilities and ensuring that the sum of probabilities for all classes increases to 1.

#### Architecture of the LSTM + RNN model

The architecture described combines both long short-term memory (LSTM) and simple recurrent neural network (RNN) layers for emotion detection tasks, particularly focused on analyzing sequential speech signals. This combination is beneficial because each type of layer provides unique advantages to the model. The LSTM layer is known for capturing long-term dependencies in sequential data, making it suitable for tasks where understanding context over extended periods is crucial. On the other hand, RNN layers provide a simpler recurrent structure that can complement the capabilities of LSTM. Although RNNs may struggle to capture long-term dependencies compared to LSTMs, they can still contribute by capturing shorter-term patterns and aiding in the overall learning process.

The model starts with an LSTM layer consisting of 256 units. This layer is configured to return sequences of outputs for each time step, allowing it to capture long-term dependencies in the input data. The LSTM layer used here can be represented as:

25 $$\:LSTM\: :=\:LSTM(256,input\_shape\:=\left({x}_{tr}.shape\left[1\right],\: {x}_{tr}. shape\left[2\right]\right),\:return\_sequences\:\:=True)$$

Here, $\:{x}_{train}.shape\left[1\right]$ represents the number of columns in feature matrix $\:{\boldsymbol{x}}_{\boldsymbol{t}\boldsymbol{r}\boldsymbol{a}\boldsymbol{i}\boldsymbol{n}}$, and $\:{x}_{train}.shape\left[2\right]$ represents the number of dimensions in feature matrix $\:{\boldsymbol{x}}_{\boldsymbol{t}\boldsymbol{r}\boldsymbol{a}\boldsymbol{i}\boldsymbol{n}}$. The dimensions of the feature matrix $\:x\_train$ determine the input shape, ensuring compatibility with the input data structure.

The LSTM layer is a dense layer with 128 neurons and a rectified linear unit (ReLU) activation function. This dense layer is fully connected to the preceding LSTM layer, enabling the model to learn complex representations based on the temporal features captured by the LSTM. A dropout layer with a dropout rate of 0.3 is applied after the dense layer to prevent overfitting. This dropout regularization randomly drops 30% of the units’ outputs during training, promoting better generalization and reducing the risk of overfitting. Next, an RNN layer with 128 units is added to provide a simpler recurrent structure that complements the capabilities of the LSTM. This layer helps capture additional temporal dynamics in the data. The RNN layer is another dense layer with 64 neurons and ReLU activation, further enhancing the model’s ability to learn representations from the combined features captured by the LSTM and RNN layers. To continue regularization, dropout layers with a rate of 0.3 are applied after each dense layer. These dropout layers help prevent overfitting by randomly dropping 30% of the units’ outputs during training. An additional dense layer with 64 neurons and ReLU activation is added for further representation learning, followed by dropout layers with a rate of 0.2 applied to both dense layers for additional regularization. Finally, the output layer consists of neurons equal to the number of emotion classes in the dataset, and the SoftMax activation function is used to generate class probabilities. This output layer produces the final predictions for emotion classification based on the learned representations from the preceding layers.

**Fig. 8:**
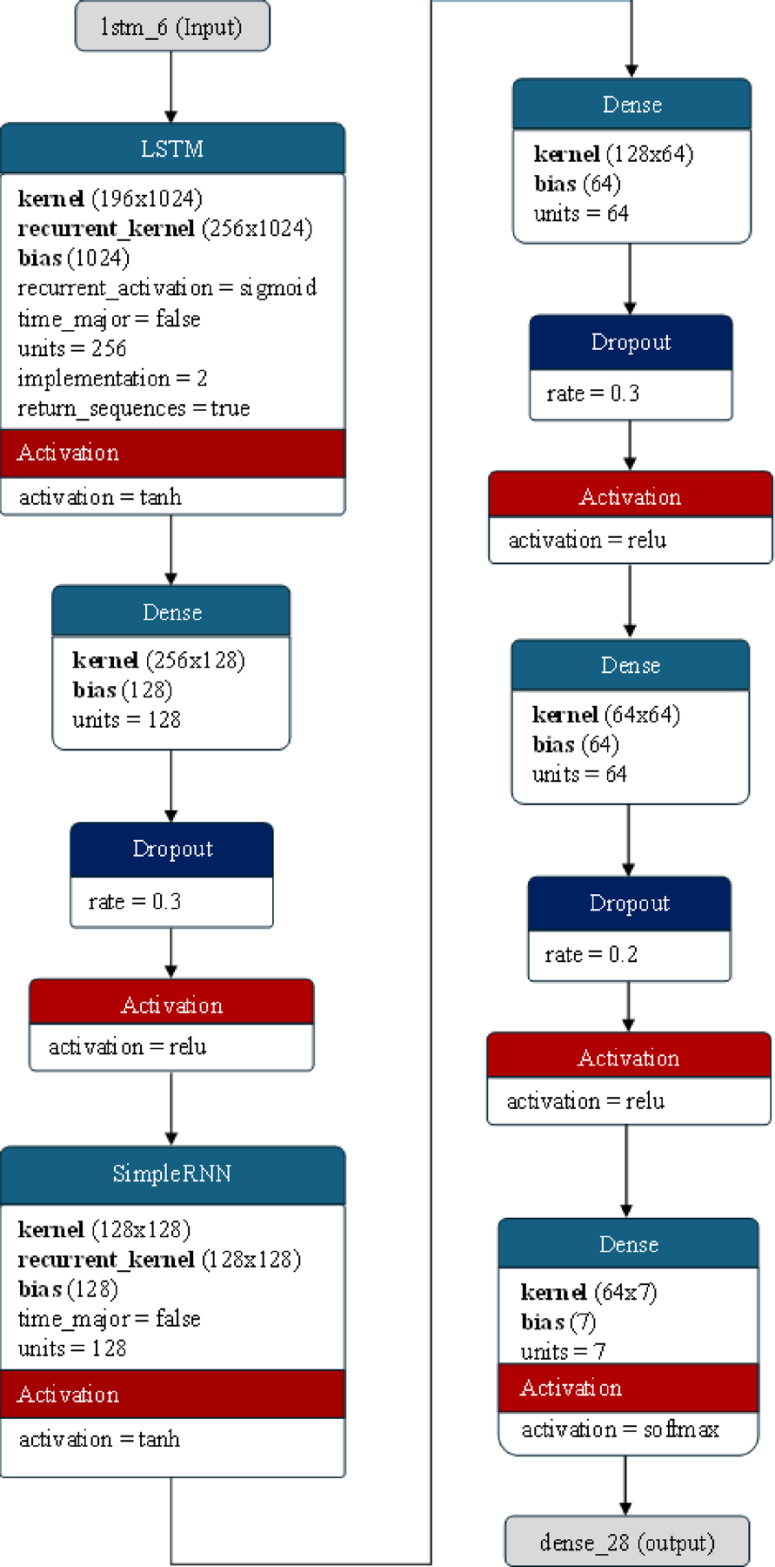
The architecture of the LSTM + RNN hybrid model used for emotion detection.

The architecture of the LSTM and RNN hybrid is presented in Fig. 8.

#### Architecture of the CNN + LSTM model

**Fig. 9:**
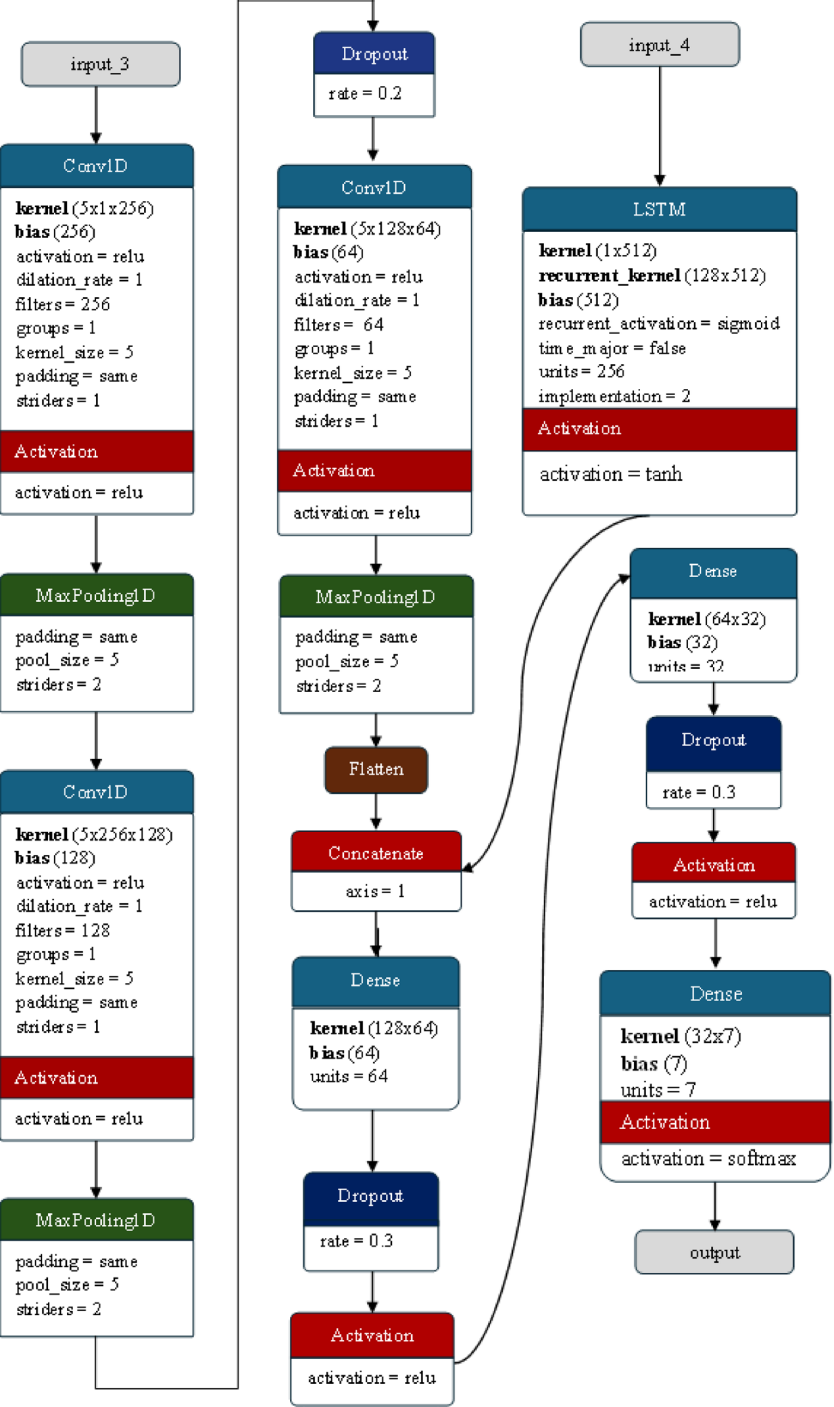
The architecture of the CNN + LSTM hybrid model used for emotion detection.

Combining a convolutional neural network (CNN) and a long short-term memory (LSTM) network in a single model for emotion detection from voice signals offers several significant advantages. CNNs are proficient at capturing spatial features, making them well suited for extracting patterns from localized regions within the input data. In the context of voice signals, where emotions are often conveyed through subtle variations in pitch, tone, and spectral characteristics, CNNs can identify these specific features and their spatial relationships, thereby enhancing the model’s ability to understand the nuances in emotional expression. On the other hand, LSTMs excel in capturing temporal dependencies and long-range sequential patterns. Emotions in speech are often characterized by changes over time, such as shifts in emotional intensity or cadence, which LSTMs are well suited to capture. By combining CNNs and LSTMs, the model can benefit from spatial and temporal feature extraction, allowing it to analyze sequential speech signals comprehensively. This holistic approach ensures that the model can capture local patterns and long-term dependencies crucial for accurate emotion detection. The integration of CNNs and LSTMs also enables the model to learn hierarchical representations of features. CNNs preprocess the input data by extracting spatial features, and LSTMs process the temporally structured information to capture sequential patterns. The architecture of the CNN and LSTM hybrid is presented in Fig. 9.

The hybrid CNN-LSTM model for emotion detection from voice signals incorporates a series of interconnected layers, each with distinct functionalities, to process and extract meaningful information from the input data. At the forefront of this architecture is the input layer, which serves as the entry point for the preprocessed speech signal data. Both the CNN and LSTM parts of the model receive data shaped as (number_of_time_steps, 1), where the temporal dimension represents the sequence of audio frames, and the feature dimension encapsulates the characteristics of each frame. Moving into the convolutional layers of the CNN, the model begins its spatial feature extraction process. The initial Conv1D layer employs 256 filters with a kernel size of 5, strategically designed to identify localized patterns within the input data, such as pitch variations and spectral features crucial for understanding emotions conveyed through voice. Along with the ReLU activation function, this layer introduces nonlinearity, enabling the model to learn complex relationships and hierarchical representations from the spatial features extracted by the filters. Following each Conv1D layer, MaxPooling Layers are strategically placed to downsample the feature maps, retaining essential information while reducing computational complexity. This process aids in capturing the most salient spatial features while discarding redundant details, promoting efficiency in subsequent layers. Moreover, incorporating dropout layers with a dropout rate of 0.2 after the convolutional layers acts as a regularization mechanism, mitigating overfitting by randomly deactivating some units during training, thereby enhancing the model’s generalization capabilities.

Simultaneously, the LSTM layer within the model captures temporal dependencies and long-range sequential patterns in the voice data. With 128 units dedicated to this task, the LSTM layer excels at recognizing nuanced changes over time, such as shifts in emotional intensity or cadence, contributing significantly to the model’s understanding of temporal dynamics in emotional expression. As the model progresses, the outputs from the CNN and LSTM branches are concatenated, merging spatial and temporal features into a cohesive representation through the concatenate layer. This amalgamation of spatial and temporal information enriches the model’s comprehension of emotional nuances, allowing it to analyze sequential speech signals comprehensively. Subsequent dense layers further refine the learned features, while dropout layers with a dropout rate of 0.3 safeguard against overfitting, promoting robustness and generalization in the model.

Finally, the output layer, which is tailored to the specific number of emotion classes in the dataset, employs the softmax activation function to generate class probabilities. These probabilities indicate the likelihood of each emotion class given the input voice signal, culminating in the model’s ability to detect and classify emotions conveyed through voice signals accurately.

### Stacked convolutional network (SCoNN)

The stacked convolutional network (SCoNN) is a specialized deep convolutional neural network architecture that excels in the domain of emotion detection from sequential speech signals. The nomenclature ‘Stacked’ signifies the layering of convolutional blocks, each designed to capture and refine features at various levels of abstraction, thereby enhancing the model’s ability to discern intricate patterns within the data. This stacking approach is advantageous over traditional CNNs because it provides a deeper and more nuanced analysis of sequential data, which is often required for the complex task of emotion detection. Unlike RNNs and LSTMs, which are tailored for sequence data but may struggle with long-term dependencies and computational intensity, the SCoNN leverages the innate spatial hierarchy in speech signals through its convolutional layers, thus offering a more efficient and scalable solution.

The architecture begins with a Conv1D layer equipped with 128 filters, each with a kernel size of 8 and ‘same’ padding, ensuring that the dimensionality of the output matches the input. This layer operates on the inputShape, which is the dimension of the input feature, applying the following formula:

26 $$\:Conv1D\left(X\right)=ReLU\left(\sum\:_{i=1}^{128}{W}_{i}*X+{b}_{i}\right)$$

where $\:X$ is the input, $\:{W}_{i}$ represents the weights of the$\:\:{i}_{th}$ filter, $\:{b}_{i}$ is the bias term, and $\:ReLU$ is the activation function that introduces nonlinearity.

Following each convolutional operation, batch normalization is applied and is governed by the following equation:

27 $$\:BatchNormalization\left(X\right)=\gamma\: . \frac{\left(X-\mu\:\right)}{\sqrt{{\sigma\:}^{2}+\epsilon}}+\beta\:$$

where $\:X$ is the input feature map, $\:\mu\:$ and $\:{\sigma\:}^{2}$ are the minibatch mean and variance, respectively, $\:\gamma\:$ and $\:\beta\:$ are learnable parameters and $\epsilon$ is a small constant for numerical stability.

To combat overfitting, a dropout layer with a rate of 0.5 follows, randomly nullifying half of the activations and effectively simplifying the model during training. The MaxPooling1D layer then reduces the spatial size of the representation, preserving the most significant features and reducing the number of parameters and computations in the network:

28 $$\:MaxPooling1D\left(X\right)=max\left({X}_{i:i+pool\_size}\right)$$

where $\:X$ is the input feature map and $\:pool\_size$ is the size of the window over which the maximum is computed.

**Fig. 10:**
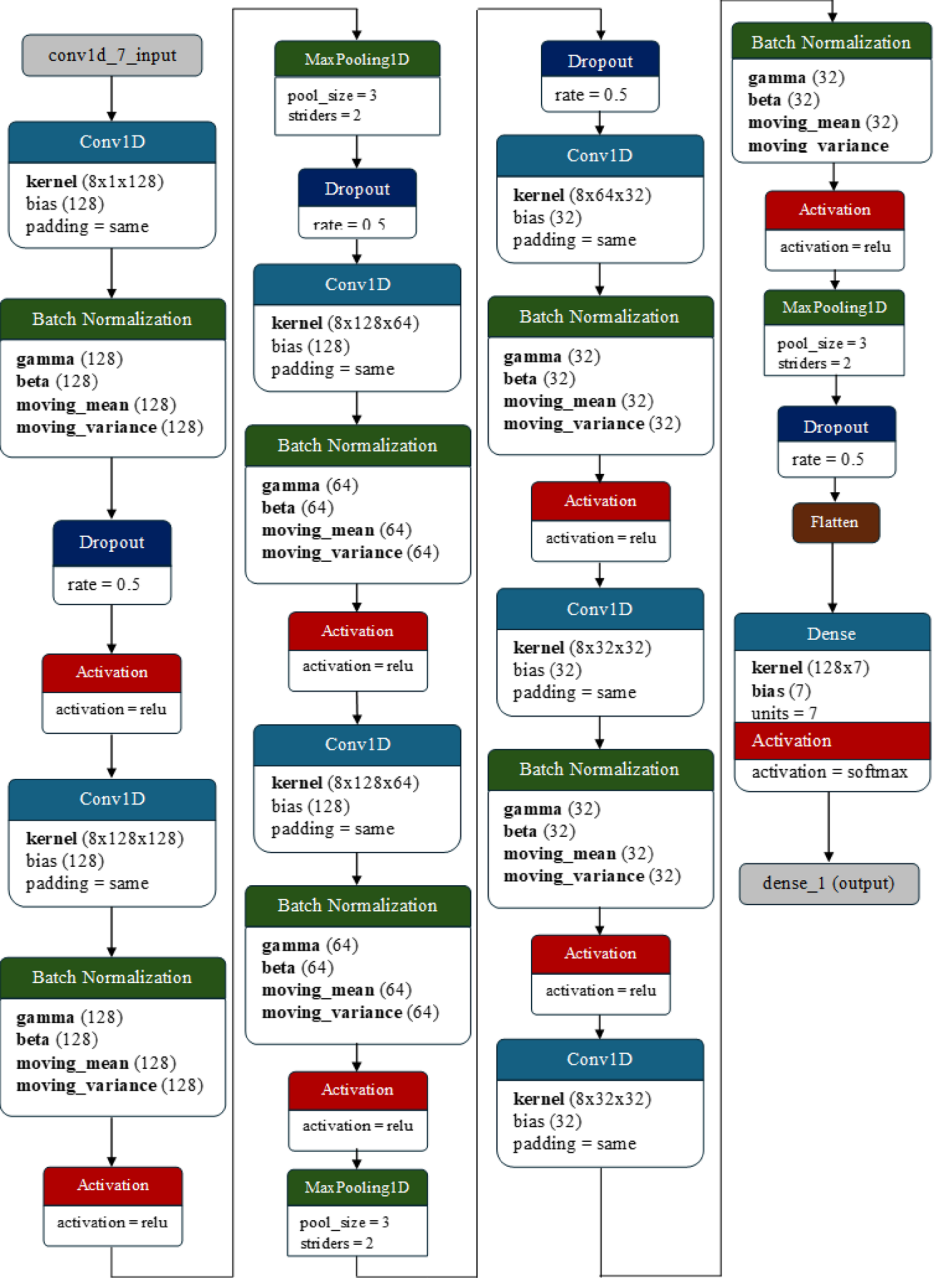
The architecture of the stacked convolutional network (SCoNN) model used for emotion detection.

The final stage of the model is a flatten layer that reshapes the feature maps into a one-dimensional vector, followed by a dense layer that maps the learned features to the output classes using the softmax activation function:

29 $$\:Dense\left(X\right)=softmax\left(W.X+b\right)$$

where $\:W$ and $\:b$ are the weights and biases of the dense layer, respectively, and the softmax function ensures that the output is a probability distribution over the classes. The architecture of the proposed SCoNN model is presented in Fig. 10.

The design of the SCoNN is suitable for emotion detection because it is believed to capture tiny differences in speech patterns that indicate different emotions. Its deep architecture and the sequential application of convolutional, normalization, and dropout layers enable it to learn complex features at multiple scales, making it highly adept at recognizing emotional context from speech signals. This includes sensitivity to both the content and the tone of spoken words. The use of 1D convolutions is specifically tailored for processing time-series data such as speech, making the SCoNN a powerful and effective tool for this application. Its architecture stands as a testament to the evolution of deep learning models, building upon the foundations of CNNs, RNNs, and LSTMs and pushing the boundaries of what is achievable in emotion detection from speech.

The Stacked Convolutional Network (SCoNN) differs from conventional CNN models through its hierarchical stacking design and adaptive feature fusion strategy. Unlike typical Conv1D-based CNNs that operate within a fixed receptive field, SCoNN employs a multi-scale stacking mechanism where each convolutional block refines the temporal resolution of features extracted from speech. This structure allows the model to capture both short-term transitions and long-term dependencies in voice modulation that are characteristic of different emotional states. Each convolutional block in SCoNN combines batch normalization and dropout in an alternating configuration, which stabilizes the feature distribution across layers and reduces internal covariate shift during training.

Another distinctive aspect of SCoNN is its adaptive feature fusion at the dense layer stage. Features extracted from the final convolutional stack are combined with complementary spectral representations such as MFCCs and Mel spectrograms. This enables the model to learn a richer joint representation that captures both temporal and frequency-based emotional cues. Traditional CNNs typically process a single feature domain, whereas SCoNN unifies multiple acoustic domains within the same network. The integration of hierarchical convolutional stacks, alternating normalization–dropout configuration, and multi-domain feature fusion gives SCoNN a unique architecture that enhances emotion recognition accuracy and generalization across datasets.

## Results & discussion

To conduct the experiment, a notebook equipped with a Windows 11 operating system, an AMD Ryzen 5 4600 H processor, 24 GB of RAM, and an Nvidia GTX 1650 DDR6 GPU with 4 GB of VRAM were used. The experiment utilized the Python programming language and various libraries, such as Librosa for audio processing, NumPy for mathematical and statistical functions, Seaborn and Matplotlib for visualization, and Scikit-Learn for preprocessing tasks. The CNN architecture was fully constructed using deep neural networks, including LSTM, CNN, and RNN, provided by TensorFlow. Spyder and Jupyter Notebooks served as integrated development environments (IDEs). A TensorFlow GPU was used to accelerate the training process of the neural network.

 The experimental process rigorously tested and analyzed various features using a range of deep learning models discussed in Sect. 3. The sensitivity, specificity, and validation accuracy metrics were calculated in detail for each model. The approach to generating results was methodically structured into distinct phases for comprehensive evaluation. All models underwent rigorous training and validation using different feature sets to estimate their performance. Subsequent phases involved individual training using specific feature subsets to understand their impact. A thorough analysis of the model’s performance metrics accompanied each iteration to discern the influence of different feature combinations on classification outcomes. The datasets were meticulously divided for robust evaluation, and the experimental setup progressed systematically, evaluating each model configuration to discern their strengths and weaknesses in emotion detection tasks.

The main goal of these experiments was to discover the hidden potential of each model architecture and determine which features contributed most significantly to improved accuracy in emotion detection. The detailed analysis and interpretation of the experimental results are explained in the following sections, which offer valuable insights into the effectiveness of different combinations of models and features.

### Results of the existing deep learning model

In this section, an exhaustive evaluation of the deep learning models outlined in Sect. 3.4 is conducted, focusing on the derived feature segments. Specifically, the assessment included 40 MFCC features, 13 LPC features, 128 Mel spectrogram features, and 15 Chroma and other features. A total of 12 chroma features, 1 RMS feature, 1 ZCR feature, and 1 pitch frequency feature constitute 15 chroma and other features. In addition, a detailed analysis is performed by testing all the deep learning models using a combination of 196 derived features. The dataset instances are partitioned, with 80% designated for training and 20% for testing.

The classification accuracy of the deep learning models evaluated on the TESS dataset is detailed in Table 3.

**Table 3:** Classification accuracy of the deep learning models on the TESS dataset.

Feature Sets	CNN + LSTM	CNN	LSTM	RNN + LSTM
Combined (196)	99.64	99.75	99.71	99.82
MFCC (40)	99.57	99.75	99.96	99.85
LPC (13)	91.78	98.18	92.93	88.00
Mel Spectrogram (128)	98.93	98.21	99.10	98.68
Chroma & Others (15)	81.64	77.82	83.50	81.40

The analysis of deep learning models on the TESS dataset, as delineated in Table 3, demonstrated substantial classification accuracy across diverse feature sets. When employing the combined feature set comprising 196 features, all models attained exceptional accuracy, with the RNN + LSTM model marginally surpassing the others at 99.82%. Notably, the utilization of MFCC features (40) yielded sustained high accuracy, with the LSTM model excelling at 99.96%, underscoring its ability to capture temporal dynamics from these features. Conversely, the performance of models using LPC features (13) exhibited significant variability, with the CNN model achieving the highest accuracy at 98.18%, while the RNN + LSTM model lagging at 88.00%. Furthermore, the accuracy remained robust across models when utilizing Mel Spectrogram features (128), with the LSTM model achieving the highest accuracy at 99.10%. However, for Chroma and other features (15), the models generally demonstrated less effectiveness, with the LSTM model once again leading at 83.50%. This suggests that the LSTM model has a consistent ability to handle various feature types while highlighting the challenges associated with less informative features.

**Table 4:** Classification accuracy of the deep learning models on the RAVDESS datasets.

Feature Sets	CNN + LSTM	CNN	LSTM	RNN + LSTM
Combined (196)	82.21	88.69	87.72	86.31
MFCC (40)	80.36	81.02	90.48	90.84
LPC (13)	77.68	73.36	60.79	59.67
Mel Spectrogram (128)	81.10	81.02	79.09	79.02
Chroma & Others (15)	33.63	32.07	30.88	29.91

The classification accuracies of the deep learning models on the RAVDESS dataset, as outlined in Table 4, are more variable and generally lower than those on the TESS dataset. For the combined feature set of 196 features, the CNN model achieved the highest accuracy at 88.69%, while the CNN + LSTM model performed the lowest at 82.21%. With MFCC features (40), the LSTM and RNN + LSTM models performed similarly well, with accuracies of 90.48% and 90.84%, respectively, suggesting that these models effectively leveraged temporal information from MFCC features. The LPC features (13) resulted in significantly lower accuracies, especially for the LSTM and RNN + LSTM models, which achieved only 60.79% and 59.67%, respectively, indicating a challenge in learning from these features. For the Mel Spectrogram features (128), the accuracies were relatively consistent across the models, with a slight edge to the CNN model at 81.02%. The Chroma and other features (15) resulted in the poorest performance, with accuracies dropping significantly across all models, with the highest being just 33.63% for the CNN + LSTM model, reflecting the limited discriminative power of these features.

Comparing the models’ behaviors between the TESS and RAVDESS datasets, it is evident that the TESS dataset allowed for higher classification accuracies overall, particularly with combined features and MFCCs. The decrease in performance on the RAVDESS dataset across all feature sets indicates that this dataset presents more complexity or variability that the models struggle to capture as effectively as with the TESS dataset. Notably, while the LSTM-based models excelled with MFCC features in both datasets, they showed a marked decrease in accuracy with LPC features on the RAVDESS dataset, highlighting dataset-specific challenges in feature representation and model adaptation.

**Table 5:** Classification accuracy of the deep learning models on the RAVDESS datasets.

Feature Sets	CNN + LSTM	CNN	LSTM	RNN + LSTM
Combined (196)	91.67	87.38	90.95	90.48
MFCC (40)	88.81	88.57	90.00	91.43
LPC (13)	22.86	30.84	21.43	24.76
Mel Spectrogram (128)	88.57	92.86	86.19	89.76
Chroma & Others (15)	49.52	54.76	46.43	48.57

The classification accuracy of the deep learning models on the SAVEE dataset, as presented in Table 5, varies based on the feature sets utilized. For the combined feature set of 196 features, the CNN + LSTM model achieved the highest accuracy at 91.67%, indicating its strong ability to integrate multiple features for better classification. The LSTM and RNN + LSTM models also performed well, with 90.95% and 90.48%, respectively, while the CNN model had a lower accuracy of 87.38%. For MFCC features (40), all models performed well, with the RNN + LSTM model achieving the highest accuracy at 91.43%, demonstrating its effectiveness in capturing temporal patterns within these features. The CNN and LSTM models also achieved strong performances, with 88.57% and 90.00% accuracy, respectively. The LPC features (13) resulted in significantly lower accuracies for all models, with the CNN model performing best at 30.84%, indicating the models’ difficulty in extracting meaningful information from these features. The LSTM model had the lowest accuracy at 21.43%, reflecting its struggle with these features. For the Mel Spectrogram features (128), the CNN model achieved an accuracy of 92.86%, highlighting its ability to process detailed spectral information. The other models also performed well, with accuracies close to 90%. With Chroma and other features (15), the models showed moderate performance, with the CNN model achieving the highest accuracy at 54.76%. This suggests that while these features are less informative, the CNN model can still extract useful patterns.

 A detailed sensitivity and specificity performance comparison of various deep learning modules used here are presented in Tables 6, 7 and 8 for the TESS, RAVDESS and SAVEE datasets, respectively.

**Table 6:** Specificity and sensitivity analysis of deep learning models on TESS dataset.

Sensitivity	neutral	1	1	0.9	1	0.99	1	1	0.99	1	0.99	1	1	0.92	1	1	1	1	0.91	1	0.99
	happy	0.99	0.99	0.86	0.99	0.71	1	1	0.99	0.95	0.62	0.99	1	0.97	1	0.81	1	1	0.88	0.98	0.77
	angry	1	1	0.98	1	0.83	1	1	1	1	0.68	1	1	0.99	1	0.8	1	1	0.97	1	0.86
	fearful	1	1	0.96	1	0.82	1	1	1	0.99	0.88	1	1	0.89	0.99	0.88	1	1	0.87	0.99	0.85
	disgust	0.99	0.97	0.93	0.99	0.71	0.99	0.99	0.95	0.96	0.67	0.99	1	0.82	1	0.67	1	1	0.66	0.97	0.63
	sad	1	1	0.98	0.99	0.89	1	1	1	1	0.87	1	1	0.99	1	0.94	1	1	0.98	1	0.9
	surprised	0.99	1	0.83	0.95	0.76	1	1	0.96	0.98	0.76	1	1	0.91	0.97	0.77	1	1	0.95	0.97	0.71
**Specificity**	**neutral**	1	1	0.99	1	1	1	1	1	1	1	1	1	1	1	1	1	1	0.98	1	1
	**happy**	1	1	0.99	0.99	0.96	1	1	0.99	1	0.96	1	1	1	1	0.94	1	1	1	1	0.95
	**angry**	1	1	1	1	0.96	1	1	1	1	0.98	1	1	1	1	1	1	1	0.99	1	0.95
	**fearful**	1	1	0.98	1	0.87	1	1	1	1	0.97	1	1	1	1	0.98	1	1	0.99	1	0.97
	**disgust**	1	1	0.97	0.99	0.95	1	1	1	1	0.93	1	1	0.99	1	0.96	1	1	0.99	1	0.96
	**sad**	1	1	1	1	1	1	1	1	1	0.99	1	1	1	1	0.99	1	1	1	1	0.99
	**surprised**	1	1	0.97	1	0.94	1	1	0.99	0.99	0.91	1	1	1	0.99	0.96	1	1	0.92	0.99	0.96
**Feature Sets**		Combined (196)	MFCC (40)	LPC (13)	Mel Spectrogram (128)	Chroma & Others (15)	Combined (196)	MFCC (40)	LPC (13)	Mel Spectrogram (128)	Chroma & Others (15)	Combined (196)	MFCC (40)	LPC (13)	Mel Spectrogram (128)	Chroma & Others (15)	Combined (196)	MFCC (40)	LPC (13)	Mel Spectrogram (128)	Chroma & Others (15)
**Deep Learning**		CNN + LSTM					CNN					LSTM					RNN + LSTM				
**Methods**																					

**Table 7:** Specificity and sensitivity analysis of deep learning models on RAVDESS dataset.

Sensitivity	angry	0.82	0.88	0.9	0.88	0.42	0.9	0.77	0.85	0.86	0.42	0.91	0.92	0.74	0.86	0.49	0.91	0.92	0.76	0.88	0.45
	calm	0.9	0.91	0.86	0.89	0.36	0.94	0.93	0.81	0.89	0.43	0.94	0.95	0.79	0.85	0.5	0.93	0.93	0.82	0.86	0.54
	disgust	0.82	0.72	0.8	0.85	0.34	0.86	0.86	0.77	0.8	0.32	0.86	0.91	0.63	0.81	0.16	0.83	0.94	0.6	0.81	0.16
	fearful	0.77	0.79	0.6	0.78	0.4	0.93	0.8	0.68	0.75	0.28	0.82	0.9	0.44	0.78	0.33	0.84	0.89	0.45	0.78	0.31
	happy	0.81	0.8	74	0.79	0.18	0.83	0.74	0.7	0.82	0.21	0.88	0.84	0.6	0.74	0.09	0.86	0.86	0.59	0.74	0.11
	sad	0.66	0.71	0.74	0.73	0.25	0.83	0.73	0.6	0.7	0.19	0.8	0.88	0.52	0.67	0.18	0.76	0.87	0.49	0.69	0.22
	surprised	0.84	0.81	0.76	0.78	0.44	0.92	0.84	0.74	0.85	0.39	0.92	0.94	0.53	0.8	0.4	0.92	0.94	0.48	0.75	0.3
**Specificity**	**angry**	0.97	0.96	0.94	0.97	0.88	0.97	0.99	0.95	0.97	0.84	0.98	0.98	0.92	0.96	0.86	0.98	0.98	0.94	0.97	0.81
	**calm**	0.97	0.97	0.97	0.98	0.93	0.99	0.97	0.95	0.98	0.87	0.98	0.99	0.93	0.97	0.79	0.97	0.98	0.91	0.97	0.77
	**disgust**	0.94	0.97	0.97	0.95	0.86	0.98	0.94	0.96	0.97	0.86	0.98	0.99	0.95	0.95	0.94	0.98	0.98	0.95	0.95	0.84
	**fearful**	0.98	0.96	0.97	0.95	0.91	0.97	0.97	0.94	0.96	0.95	0.99	0.99	0.95	0.97	0.91	0.98	0.99	0.93	0.97	0.91
	**happy**	0.96	0.95	0.98	0.98	0.9	0.99	0.97	0.97	0.97	0.92	0.98	0.98	0.93	0.98	0.95	0.98	0.98	0.94	0.97	0.94
	**sad**	0.97	0.97	0.95	0.96	0.92	0.98	0.97	0.97	0.96	0.92	0.97	0.98	0.93	0.96	0.93	0.98	0.98	0.93	0.96	0.91
	**surprised**	0.97	0.98	0.96	0.99	0.82	0.97	0.96	0.95	0.96	0.84	0.98	0.98	0.93	0.96	0.81	0.98	0.99	0.94	0.96	0.85
**Feature Sets**		Combined (196)	MFCC (40)	LPC (13)	Mel Spectrogram (128)	Chroma & Others (15)	Combined (196)	MFCC (40)	LPC (13)	Mel Spectrogram (128)	Chroma & Others (15)	Combined (196)	MFCC (40)	LPC (13)	Mel Spectrogram (128)	Chroma & Others (15)	Combined (196)	MFCC (40)	LPC (13)	Mel Spectrogram (128)	Chroma & Others (15)
**Deep Learning**		CNN + LSTM					CNN					LSTM					RNN + LSTM				
**Methods**																					

**Table 8:** Specificity and sensitivity analysis of deep learning models on SAVEE dataset.

Sensitivity	angry	0.92	0.91	0.56	0.84	0.41	0.91	0.88	0.5	0.9	0.42	0.95	0.94	0.53	0.78	0.33	0.92	0.92	0.53	0.82	0.39
	disgust	0.86	0.91	0.86	0.86	0.31	0.91	0.89	0.42	0.92	0.5	0.93	0.96	0.21	0.88	0.21	0.89	0.95	0.21	0.93	0.21
	fearful	0.9	0.88	0.12	0.95	0.44	0.73	0.87	0.17	0.9	0.46	0.84	0.84	0.16	0.82	0.37	0.92	0.85	0.13	0.9	0.52
	happy	0.84	0.9	0	0.88	0.6	0.86	0.86	0.12	0.9	0.57	0.88	0.9	0	0.84	0.62	0.88	0.9	0.7	0.84	0.59
	neutral	0.98	0.98	0.15	0.96	0.79	0.98	0.91	0.42	0.94	0.58	0.97	0.94	0.39	0.97	0.77	0.96	0.97	0.52	0.97	0.79
	sad	0.95	0.84	0.6	0.84	0.69	0.94	0.94	0.18	0.97	0.71	0.95	0.9	0.26	0.93	0.7	0.95	0.97	0.24	0.97	0.72
	surprised	0.96	0.76	0.42	0.85	0.23	0.8	0.84	0.31	0.96	0.47	0.84	0.83	0	0.78	0.2	0.91	0.84	0.62	0.83	0.16
**Specificity**	**angry**	0.98	0.99	0.68	0.99	0.88	0.98	0.99	0.85	0.99	0.9	0.99	0.98	0.69	0.98	0.9	0.98	98	0.68	0.98	0.89
	**disgust**	0.99	0.99	0.91	0.98	0.94	0.98	0.98	0.87	0.99	0.93	0.99	0.99	0.9	0.98	0.94	0.99	0.99	0.92	0.98	0.84
	**fearful**	0.98	0.97	0.9	0.98	0.94	0.97	0.97	0.9	1	0.97	0.97	0.98	0.89	0.96	0.9	0.97	0.98	0.92	0.99	0.88
	**happy**	1	0.97	1	0.96	0.9	0.97	0.98	0.92	0.99	0.91	0.97	0.98	0.99	0.98	0.88	0.96	0.98	0.96	0.98	0.9
	**neutral**	0.99	0.98	0.96	0.97	0.94	1	0.99	0.86	0.98	0.97	1	0.98	0.82	0.98	0.92	1	0.99	0.8	0.99	0.92
	**sad**	0.99	0.99	0.96	0.99	0.9	0.99	0.98	0.91	1	0.94	1	0.99	0.81	0.99	0.88	0.99	0.99	0.86	0.99	0.9
	**surprised**	0.97	0.98	0.68	0.99	0.92	0.96	0.98	0.89	0.97	0.86	0.97	0.98	1	0.97	0.96	0.99	0.98	0.98	0.97	0.97
**Feature Sets**		Combined (196)	MFCC (40)	LPC (13)	Mel Spectrogram (128)	Chroma & Others (15)	Combined (196)	MFCC (40)	LPC (13)	Mel Spectrogram (128)	Chroma & Others (15)	Combined (196)	MFCC (40)	LPC (13)	Mel Spectrogram (128)	Chroma & Others (15)	Combined (196)	MFCC (40)	LPC (13)	Mel Spectrogram (128)	Chroma & Others (15)
**Deep Learning**		CNN + LSTM					CNN					LSTM					RNN + LSTM				
**Methods**																					

From the detailed comparison of deep learning models across the TESS, RAVDESS, and SAVEE datasets, several key observations can be made regarding their performance and the influence of different feature sets on classification accuracy.

- The TESS dataset makes it easier for models to classify emotions accurately, likely due to clearer emotional patterns.
- The RAVDESS and SAVEE datasets are more challenging, resulting in lower accuracies, suggesting the need for more sophisticated models or feature engineering.
- Apart from combined features, the MFCC and Mel Spectrum features are most effective across all datasets, while LPC features are the least effective.
- CNNs have strong spectral features, while LSTM-based models excel with sequential features such as MFCCs.

### Results of the proposed stacked convolutional network (SCoNN)

A previous experiment showed that the MFCC and Mel Spectrogram features yielded the highest accuracy. Consequently, the proposed SCoNN model was evaluated using these two feature segments separately and by combining both. The experiment was conducted separately for each dataset. The datasets were split into training (80%), testing (20%), and validation (10%) sets.

During the training phase, various learning rates were tested, and a learning rate of 0.0001 produced the best results without any overfitting across all datasets. Early stopping was implemented to prevent overfitting, with the “patience” parameter set to 12 and “val_loss” being monitored. The training process was set for 500 epochs, but the early stopping mechanism was set to halt it earlier.

All key hyperparameters, including the learning rate, dropout rate, and kernel size, were optimized through systematic tuning experiments to achieve the best trade-off between accuracy and generalization.

#### Performance analysis of the sconn on the RAVDESS dataset

**Fig. 11:**
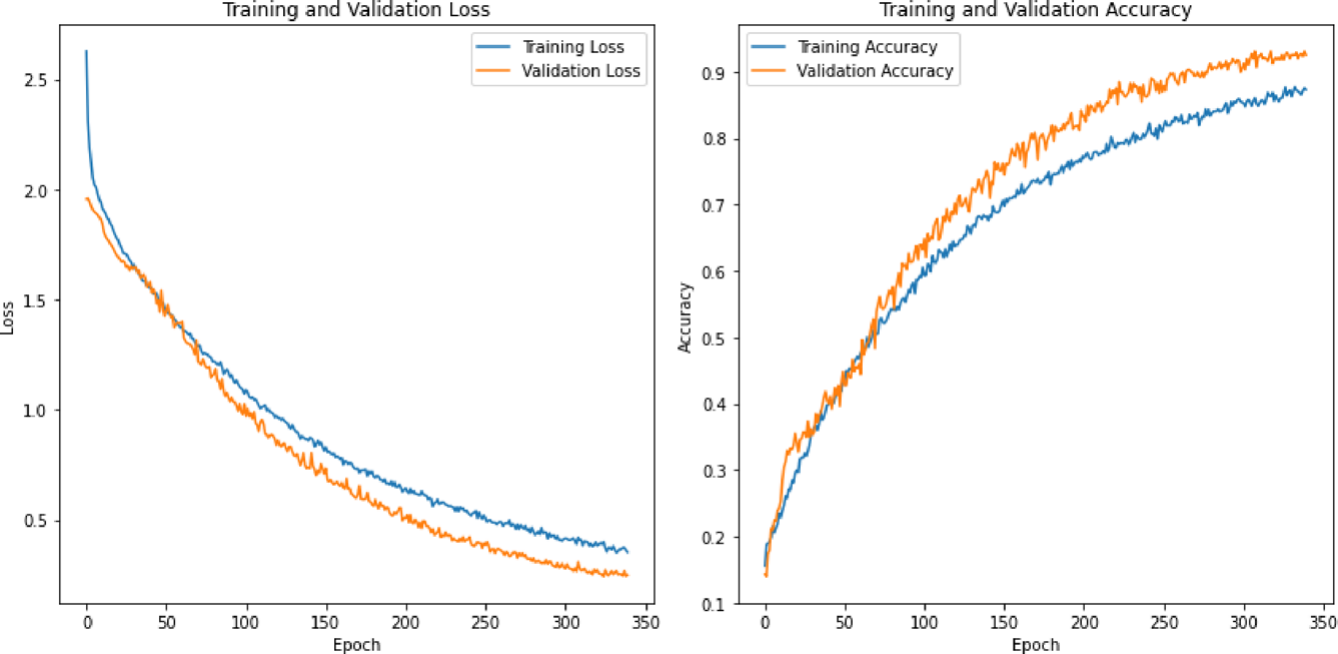
Loss and accuracy of training and validation over epochs of the SCoNN model using the Mel spectrogram of the RAVDESS dataset.

Figure 11 represents the validation vs. training loss and validation vs. training accuracy of the proposed model on the RAVDESS dataset. Here, the X-axis of both graphs represents the number of iterations over the entire training dataset, also called epochs; the Y-axis of the left-hand side graph represents the loss value; and the Y-axis of the right-hand side graph represents the accuracy of the model. The blue line represents the training loss and training accuracy for both graphs. Similarly, the orange line represents the validation loss and validation accuracy for both graphs. In this experiment, we trained our proposed model using only the Mel spectrogram. During the training process, we can clearly see that both the model’s training and validation accuracy increase over epochs, and the training and validation loss decrease over the epochs. This shows that our model performs as expected without any overfitting.

**Fig. 12:**
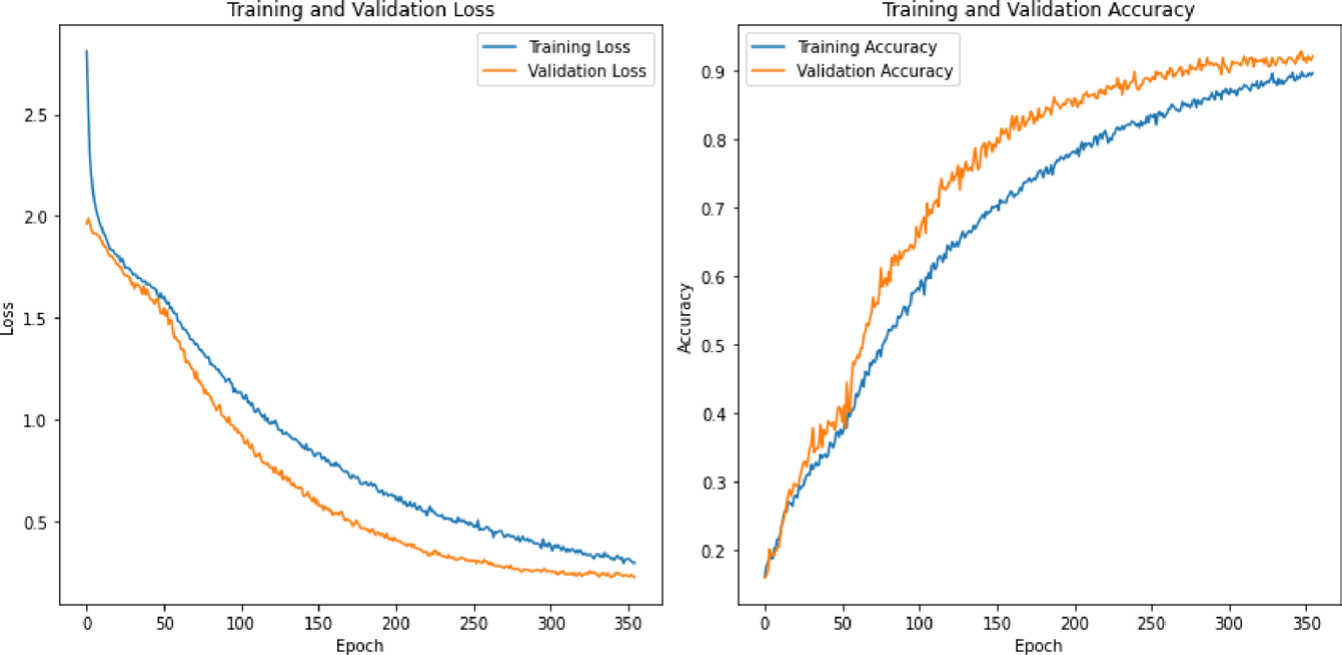
Loss and accuracy of training and validation over epochs of the SCoNN model using MFCC features of the RAVDESS dataset.

**Fig. 13:**
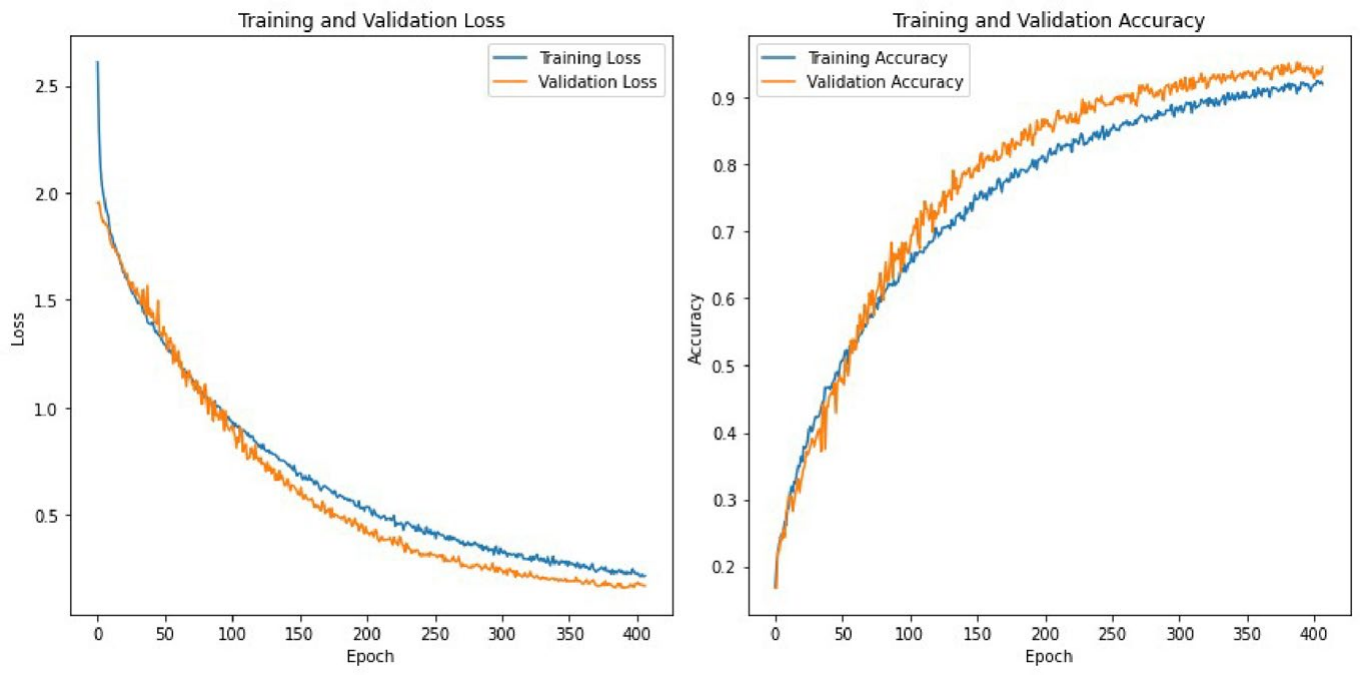
Loss and accuracy of training and validation over epochs of the SCoNN model using the Mel Spectrogram + MFCC features of the RAVDESS dataset.

Similarly, Figs. 12 and 13 demonstrate the model’s performance over epochs when trained using MFCC features and a combination of both the Mel Spectrogram and MFCC features, respectively, on the RAVDESS dataset. As shown in Figs. 11 and 12, and 13, both the training and validation losses decrease over epochs, and the training and validation accuracies increase over epochs. However, we can see that there are some fluctuations in the validation loss and accuracy, which are normal and can be due to the variability in the validation data.

#### Performance analysis of the sconn on the TESS dataset

**Fig. 14:**
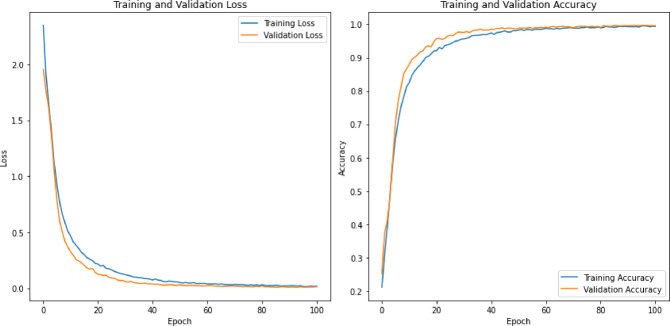
Loss and accuracy of training and validation over epochs of the SCoNN model using the Mel Spectrogram of the TESS dataset.

**Fig. 15:**
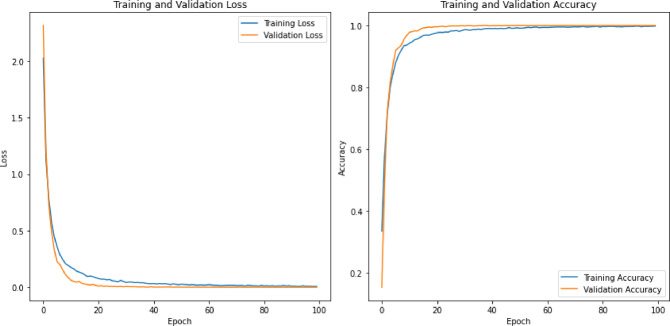
Loss and accuracy of training and validation over epochs of the SCoNN model using the MFCC of the TESS dataset.

**Fig. 16:**
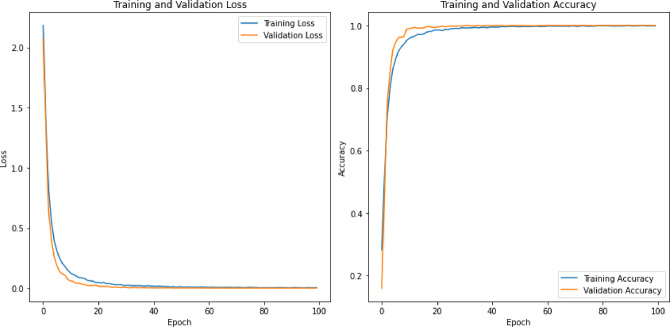
Loss and accuracy of training and validation over epochs of the SCoNN model using the MFCC + Mel Spectrogram of the TESS dataset.

Figure 14 represents the validation vs. training loss and validation vs. training accuracy of the proposed model on the TESS dataset. Here, the X-axis of both graphs represents the number of iterations over the entire training dataset, also called epochs; the Y-axis of the left-hand side graph represents the loss value; and the Y-axis of the right-hand side graph represents the accuracy of the model. The blue line represents the training loss and training accuracy for both graphs. Similarly, the orange line represents the validation loss and validation accuracy for both graphs. In this experiment, we trained our proposed model using only the Mel spectrogram. During the training process, we can clearly see that both the model’s training and validation accuracy increase over epochs, and the training and validation loss decrease over the epochs. This shows that our model performs as expected without any overfitting. Similarly, Figs. 15 and 16 demonstrate the model’s performance over epochs while trained using MFCC features and a combination of both the Mel Spectrogram and MFCC features, respectively, on the TESS dataset. As shown in Fig. 14, in Figs. 15 and 16, both the training and validation losses decrease over epochs, and the training and validation accuracies increase over epochs. Unlike in the RAVDESS dataset, no fluctuations occurred during the training process.

In every graph, there is a sharp decrease in both training and validation loss within the first 15–20 epochs, indicating that the model is quickly learning and improving its prediction. The close alignment of the training and validation curves suggested that the model generalized well to the validation set, with no signs of overfitting. Overfitting would be indicated by a significant gap between training and validation performance, which is not evident here.

In this study, the datasets were divided into 80% training, 10% validation, and 20% testing subsets to ensure a clear separation of data during training and evaluation. Early stopping with a patience value of 12, monitored on validation loss, was applied to avoid overfitting. The consistent trend and close alignment of the training and validation curves in Figs. 14, 15 and 16 confirm that the proposed SCoNN model generalized effectively across the TESS dataset without signs of overfitting or data leakage.

#### Performance analysis of sconn on the SAVEE dataset

**Fig. 17:**
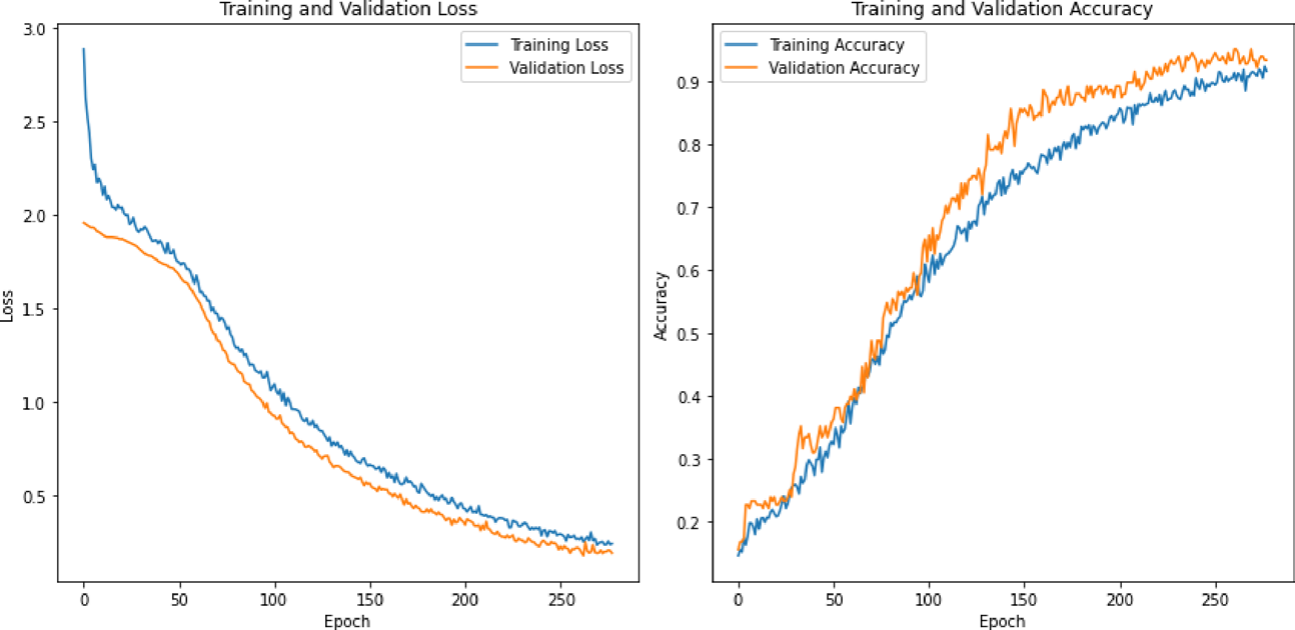
Loss and accuracy of training and validation over epochs of the SCoNN model using Mel Spectrogram features of the SAVEE dataset.

**Fig. 18:**
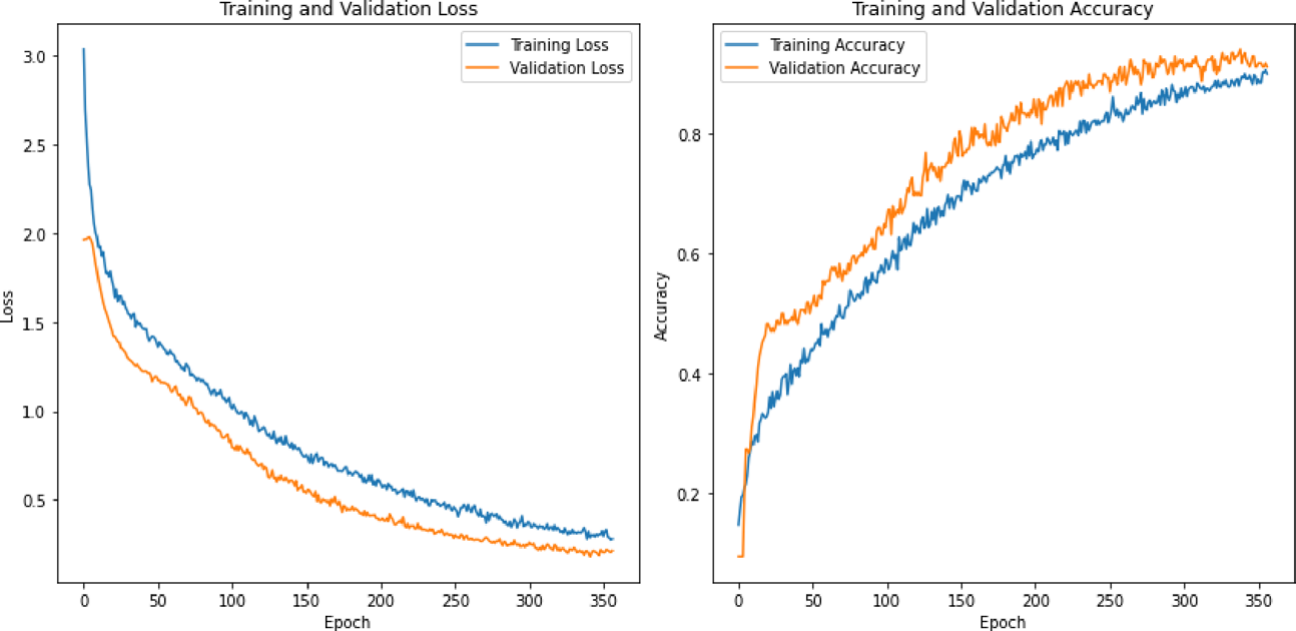
Loss and accuracy of training and validation over epochs of the SCoNN model using MFCC features on the SAVEE dataset.

**Fig. 19:**
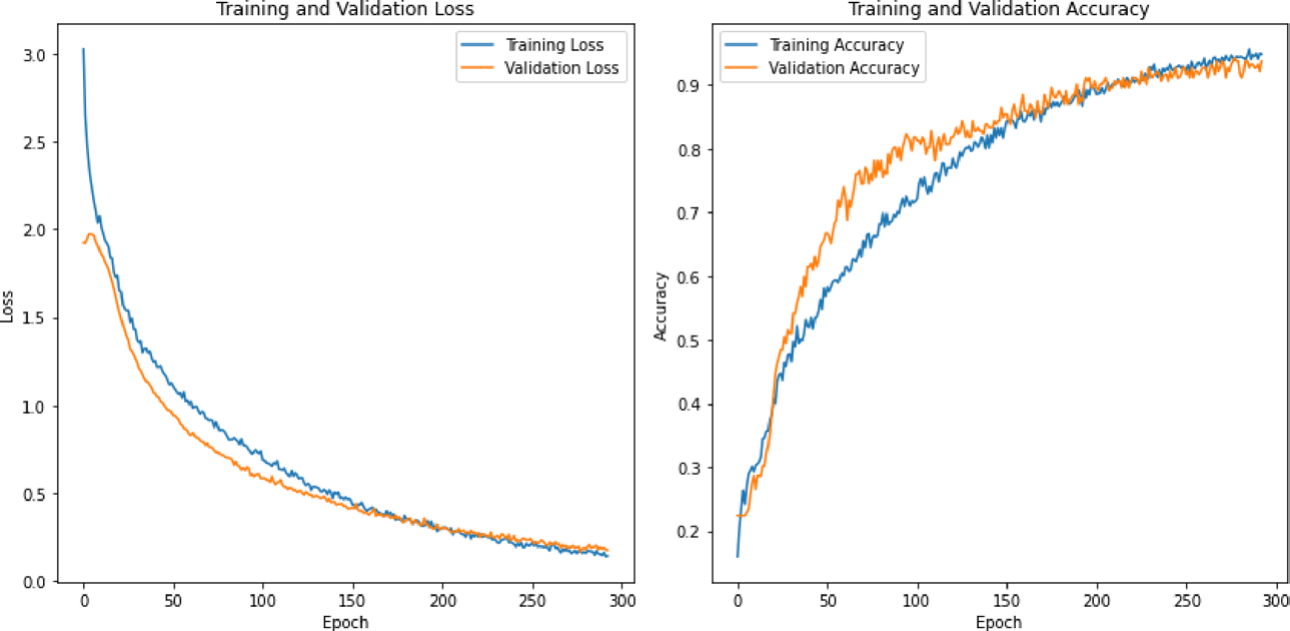
Loss and accuracy of training and validation over epochs of the SCoNN model using MFCC + Mel Spectrogram features on the SAVEE dataset.

Figure 17 represents the validation vs. training loss and validation vs. training accuracy of the proposed model on the SAVEE dataset. Here, the X-axis of both graphs represents the number of iterations over the entire training dataset, also called epochs; the Y-axis of the left-hand side graph represents the loss value; and the Y-axis of the right-hand side graph represents the accuracy of the model. The blue line represents the training loss and training accuracy for both graphs. Similarly, the orange line represents the validation loss and validation accuracy for both graphs. In this experiment, we trained our proposed model using only the Mel spectrogram. During the training process, we can clearly see that both the model’s training and validation accuracy increase over epochs, and the training and validation loss decrease over the epochs. This shows that our model performs as expected without any overfitting. Similarly, Figs. 18 and 19 demonstrate the model’s performance over epochs while trained using MFCC features and a combination of both the Mel Spectrogram and MFCC features, respectively, on the SAVEE dataset. As shown in Figs. 17 and 18, and 19, both the training and validation losses decrease over epochs, and the training and validation accuracies increase over epochs. However, we can see that there are some fluctuations in the validation accuracy, which can be due to the lower number of samples present in the dataset as well as the variability in the validation data.

#### Accuracy and loss analysis of the sconn across all datasets

The classification accuracy of the model across all the datasets using MFCC, Mel Spectrogram, and the combination of both is presented in Table 9. Similarly, the test loss of the SCoNN model is also presented in Table 10.

**Table 9:** Classification accuracy of the proposed sconn deep learning models on the RAVDESS, SAVEE and TESS datasets.

Feature Sets	RAVDESS	SAVEE	TESS
MFCC (40)	91.51%	91.43%	99.93%
Mel Spectrogram (128)	90.63%	94.76%	99.68%
MFCC (40) + Mel Spectrogram (128)	93.30%	95.00%	99.93%

Table 9 reveals the performance of the SCoNN deep learning model using 40 MFCC features and 128 Mel Spectrogram features, and a combination of both is remarkable across the RAVDESS, SAVEE, and TESS datasets, demonstrating its efficacy in emotion detection from voice signals. When employing only the MFCC features, the model achieved impressive classification accuracies of 91.51%, 91.43%, and a near-perfect 99.93% on the RAVDESS, SAVEE, and TESS datasets, respectively. This indicates that MFCC features alone are highly effective, particularly for the TESS dataset. The use of 128 Mel Spectrogram features also yielded high accuracies, with 90.63% for RAVDESS, 94.76% for SAVEE, and 99.68% for TESS, showing that Mel Spectrograms are particularly beneficial for the SAVEE dataset, where they outperformed MFCC features. Combining both feature sets, the SCoNN model achieved the highest accuracies of 93.30%, 95.00%, and 99.93% for RAVDESS, SAVEE, and TESS, respectively. This combination consistently improved over either feature set alone, enhancing the performance on the RAVDESS and SAVEE datasets. The results suggest that integrating MFCC and Mel Spectrogram features allows the model to leverage complementary information, enhancing classification accuracy and robustness across different datasets. This combined approach maximizes the model’s ability to capture diverse acoustic features crucial for accurate emotion detection, making it a highly effective strategy for voice-based emotion classification tasks.

**Table 10:** Test loss of the proposed sconn deep learning models on the RAVDESS, SAVEE and TESS datasets.

Feature Sets	RAVDESS	SAVEE	TESS
MFCC (40)	25.61%	23.66%	0.25%
Mel Spectrogram (128)	32.32%	19.50%	0.74%
MFCC (40) + Mel Spectrogram (128)	23.09%	12.70%	0.22%

However, while the SCoNN model’s classification accuracy is impressive, it also exhibits some testing loss, as outlined in Table 10. This provides further insight into its efficiency and robustness across different datasets. The testing losses for the MFCC (40) feature set were 25.61% for RAVDESS, 23.66% for SAVEE, and 0.25% for TESS. These results indicate that while the model performs extremely well on the TESS dataset with minimal loss, the higher losses on RAVDESS and SAVEE suggest some degree of overfitting or difficulty in generalizing across these datasets. Using the Mel Spectrogram (128) features, the model achieved testing losses of 32.32% for RAVDESS, 19.50% for SAVEE, and 0.74% for TESS. These results demonstrate that while Mel Spectrogram features improved the performance on the SAVEE dataset, they resulted in greater test losses on the RAVDESS and TESS datasets than did the MFCC features, indicating some trade-offs between accuracy and loss. The combination of MFCC (40) and Mel Spectrogram (128) features yielded testing losses of 23.09% for RAVDESS, 12.70% for SAVEE, and 0.22% for TESS. This combination provided not only the best accuracy but also the lowest test loss, which was particularly notable on the SAVEE dataset, where the test loss decreased significantly to 12.70%. The low test loss on TESS (0.22%) with near-perfect accuracy further highlights the model’s exceptional performance on this dataset.

In summary, the SCoNN model’s performance reflects a balance between high classification accuracy and acceptable testing loss across different feature sets and datasets. The combined feature of MFCC and Mel Spectrograms appears to be effective, enhancing the model’s generalizability and robustness, reducing testing loss, and maximizing accuracy. This indicates that integrating diverse acoustic features allows the model to capture a broader range of emotional cues, leading to superior performance and stability in voice-based emotion detection.

#### Sensitivity and specificity analysis of the sconn across all datasets

This section presents a detailed analysis of the sensitivity and specificity of the SCoNN model using the RAVDESS, SAVEE, and TESS datasets. The analysis evaluates the model’s performance with a Mel Spectrogram, MFCC features, and a combination of both feature sets. Tables 8 and 9, and 10 outline the sensitivity and specificity of the SCoNN model for each emotion class, using the different feature sets across the RAVDESS, SAVEE, and TESS datasets.

First, the sensitivity and specificity of the SCoNN model for each emotion class were evaluated using a Mel Spectrogram, MFCC, and a combination of both feature sets in the RAVDESS dataset. This analysis provides a detailed assessment of the model’s effectiveness in reliably recognizing emotions and is outlined in Table 11.

**Table 11:** Sensitivity and specificity of the sconn model for each emotion class using the mel Spectrogram, MFCC, and mel Spectrogram + MFCC features of the RAVDESS dataset.

Emotion Classes	Mel Spectrogram		MFCC		Mel Spectrogram + MFCC	
	Sensitivity	Specificity	Sensitivity	Specificity	Sensitivity	Specificity
angry	92.89%	99.13%	95.43%	98.61%	97.97%	99.04%
calm	93.33%	98.20%	98.33%	98.20%	96.67%	99.66%
disgust	91.01%	97.92%	89.42%	98.70%	92.06%	97.75%
fearful	87.21%	98.38%	84.88%	98.81%	89.53%	99.40%
happy	89.40%	99.29%	90.32%	97.96%	90.32%	98.94%
sad	84.29%	97.92%	89.01%	98.61%	90.58%	99.05%
surprised	95.96%	98.25%	92.93%	99.21%	95.96%	98.34%

The model exhibits robust performance across the majority of emotion classes when utilizing only Mel Spectrogram features. The sensitivity for the emotion “angry” is 92.89%, meaning that it accurately detects this emotion in a high percentage of cases. The specificity was 99.13%, indicating that it also accurately ruled out this emotion in a high percentage of cases. Overall, the accuracy of the emotion detection system is high for both detecting and ruling out the emotion “angry”. Furthermore, the “calm” label demonstrated a remarkable sensitivity of 93.33% and an impressive specificity of 98.20%. Nevertheless, the model exhibits a significant decrease in sensitivity in regard to emotions such as “fearful” (87.21%) and “sad” (84.29%). This indicates that the model encounters challenges in reliably recognizing these emotions based only on its Mel spectrogram characteristics.

The model demonstrates slightly distinct performance characteristics when utilizing MFCC features. The sensitivity for the emotion “angry” increased to 95.43%, while the sensitivity for the emotion “calm” increased to 98.33%. This indicates a greater level of accuracy in detecting these emotions compared to the use of Mel Spectrogram characteristics. The specificity is consistently high for all emotions, with a precision of 98.61% for “angry” and 98.20% for “calm”. Nevertheless, the sensitivity for emotions classified as “fearful” or “sad” still has potential for enhancement, as their respective scores now stand at 84.88% and 89.01%, respectively. This suggests that although MFCC features are highly efficient for specific emotions, they may not be adequate for others.

The use of both the Mel Spectrogram and the MFCC yields a performance that is well balanced and resilient. The sensitivity for the “angry” emotion was 97.97%, and the specificity was 99.04%, indicating a substantial enhancement compared to using either feature set individually. The emotion of “calm” demonstrated a remarkable level of performance, with a sensitivity of 96.67% and a specificity of 99.66%. Significantly, the sensitivity for the emotion “fearful” rises to 89.53%, and for “sad”, it climbs to 90.58%, suggesting that the combined features improve the model’s capacity to identify these emotions reliably. The specificity remained high for all emotions, with values of 99.40% for “fearful” and 99.05% for “sad”. To summarize, the sensitivity and specificity findings of the SCoNN model, as presented in Table 11, demonstrate that using both the Mel Spectrogram and MFCC features yields the best overall performance. These integrated features successfully consist of a wider array of auditory characteristics, increasing accuracy and precision for most emotional classes. The results demonstrate the model’s ability to reliably identify and distinguish between distinct emotions, establishing it as a potent tool for classifying emotions based on voice.

**Table 12:** Sensitivity and specificity of the sconn model for each emotion class using the mel Spectrogram, MFCC, and mel Spectrogram + MFCC features of the SAVEE dataset.

Emotion Labels	Mel Spectrogram		MFCC		Mel Spectrogram + MFCC	
	Sensitivity	Specificity	Sensitivity	Specificity	Sensitivity	Specificity
angry	100.00%	99.16%	92.19%	98.03%	93.65%	100.00%
disgust	93.85%	99.44%	90.77%	100.00%	92.96%	99.27%
fear	96.61%	99.45%	91.53%	98.06%	95.16%	100.00%
happy	96.55%	98.62%	84.48%	97.79%	91.07%	99.29%
neutral	92.65%	99.15%	97.06%	99.72%	95.45%	98.92%
sad	92.16%	98.37%	94.12%	99.19%	95.83%	98.61%
surprised	90.91%	99.73%	89.09%	97.26%	100.00%	98.05%

The SCoNN model’s sensitivity and specificity for each emotion class were evaluated using Mel Spectrogram, MFCC, and a combination of both feature sets on the SAVEE dataset has been outlined in Table 12. The results indicate that the model effectively detects emotions from voice signals, similar to the findings from the RAVDESS dataset. The model demonstrates outstanding performance for certain emotions when utilizing only Mel Spectrogram features on the SAVEE dataset. For example, the emotion labeled “angry” has a sensitivity of 100.00% and a specificity of 99.16% in the model. This means that the model accurately identifies and excludes instances of this emotion. Similarly, the emotions “fear” and “happy” had high sensitivity values of 96.61% and 96.55%, respectively. Additionally, their specificities exceeded 98%, indicating the strong reliability of Mel Spectrogram features in detecting these emotions. Nevertheless, the accuracy for detecting the emotion “surprised” was slightly lower at 90.91%, indicating some challenges in reliably identifying this emotion compared to others.

The model demonstrates diverse performance across distinct emotions when utilizing MFCC capabilities. The sensitivity for the “neutral” feeling was enhanced to 97.06%, while the specificity was 99.72%, showing high accuracy in detecting this emotion. Nevertheless, the sensitivity for the emotion “happy” was relatively lower at 84.48%, but the specificity remained significantly high at 97.79%. These findings suggest that while MFCC characteristics successfully capture certain emotions, they may not be as dependable for others, such as “happy” and “surprised,” which have sensitivity scores of 84.48% and 89.09%, respectively. Using the Mel Spectrogram and MFCC characteristics yields the most equitable performance across all emotional states. The sensitivity for detecting “angry” was 93.65%, with a perfect specificity of 100.00%. This shows a substantial improvement compared to using either feature set individually. The emotion of “fear” achieved a sensitivity of 95.16% and specificity of 100.00%, suggesting that the combined properties significantly improved the model’s accuracy. Similarly, the accuracy for detecting “happiness” increases to 91.07% with a precision of 99.29%, indicating superior performance compared to solely employing MFCC characteristics. The sensitivity for the emotion “surprised” achieves a perfect score of 100.00%, showcasing the model’s exceptional capability to precisely identify this emotion by utilizing a combination of characteristics.

Upon comparing these results with the RAVDESS dataset, it was observed that there are both disparities and resemblances. When analyzing the RAVDESS dataset, the combined feature set demonstrated superior performance, exhibiting high levels of sensitivity and specificity for all emotions. As an illustration, the emotion “angry” achieved a sensitivity of 97.97% and a specificity of 99.04% on the RAVDESS dataset, while it achieved 93.65% sensitivity and 100.00% specificity on the SAVEE dataset. This suggests a slightly higher level of specificity in identifying the emotion of “anger” on the SAVEE dataset. Similarly, the sensitivity for the “happy” emotion was 90.32% on the RAVDESS dataset and increased to 91.07% on the SAVEE dataset when combined features were used. This indicates a continuous and high level of performance.

In a landscape, the sensitivity and specificity results of the SCoNN model, as presented in Table 9 for the SAVEE dataset, validate that utilizing both the Mel Spectrogram and MFCC features yields the most well-rounded and resilient performance. This integrated method successfully encompasses a wider array of auditory characteristics, resulting in increased accuracy and precision for many emotional classes. The performance of the model on the SAVEE dataset demonstrates its capacity to generalize and consistently achieve high accuracy across several datasets, thereby establishing it as a robust method for emotion categorization based on voice.

**Table 13:** Sensitivity and specificity of the sconn model for each emotion class using the mel Spectrogram, MFCC, mel Spectrogram + MFCC features of the TESS dataset.

Emotion Labels	Mel Spectrogram		MFCC		Mel Spectrogram + MFCC	
	Sensitivity	Specificity	Sensitivity	Specificity	Sensitivity	Specificity
angry	99.73%	100.00%	100.00%	100.00%	100.00%	100.00%
disgust	99.51%	99.87%	100.00%	99.96%	100.00%	99.96%
fear	100.00%	100.00%	100.00%	100.00%	100.00%	100.00%
happy	99.74%	99.83%	99.74%	99.96%	99.74%	99.96%
neutral	100.00%	100.00%	100.00%	100.00%	100.00%	100.00%
sad	99.75%	100.00%	100.00%	100.00%	100.00%	100.00%
surprised	99.02%	99.92%	99.76%	100.00%	99.76%	100.00%

The SCoNN model demonstrated exceptional sensitivity and specificity in recognizing emotions from voice sounds in the TESS dataset, as presented in Table 13. This performance is consistent with the results obtained from the RAVDESS and SAVEE datasets when employing the Mel Spectrogram, MFCC, and combined feature sets. The model achieves almost perfect sensitivity and specificity for most emotions when using only Mel Spectrogram features on TESS. For example, the sensitivity for “angry” was 99.73%, and the specificity was 100.00%. Similarly, the sensitivity for “disgusting” was 99.51%, and the specificity was 99.87%. MFCC characteristics exhibit outstanding performance, achieving excellent sensitivity and specificity for various emotions such as “angry,” “fear,” “neutral,” and “sad,” all with a 100.00% accuracy rate. Integrating the Mel Spectrogram and MFCC characteristics significantly improved the model’s accuracy, resulting in excellent sensitivity and specificity for various emotions while maintaining high performance. When comparing these results to those of RAVDESS and SAVEE, TESS consistently shows greater and more consistent performance across different emotions, with fewer instances of variation. As an illustration, the RAVDESS exhibited a slightly lower sensitivity of 97.97% for the emotion “anger” and 90.32% for the emotion “happy”, but the TESS attained a perfect sensitivity of 100.00% for both emotions. Similarly, the SAVEE dataset exhibited some degree of variation, with a sensitivity of 93.65% for the emotion “anger” and 91.07% for the emotion “happy”. In contrast, the TESS dataset consistently achieved flawless or almost flawless scores for most emotions. The SCoNN model’s resilience and effectiveness in voice-based emotion classification tasks are confirmed by its consistency and high accuracy across multiple datasets. This establishes it as a trustworthy and strong tool in this domain.

The design of SCoNN was guided by empirical evaluation of several structural variants, including models with differing numbers of convolutional blocks, kernel sizes, and dropout configurations. Preliminary experiments indicated that three stacked Conv1D blocks with alternating batch normalization and dropout layers achieved the best trade-off between complexity and performance. Increasing the number of blocks led to overfitting on smaller datasets (SAVEE and RAVDESS), while fewer blocks reduced feature discrimination accuracy. Similarly, replacing batch normalization with layer normalization or removing dropout reduced overall validation accuracy by 2–3%. These observations support the adopted configuration. Although a full ablation study was not included due to space limitations, the comparative results across CNN, LSTM, and hybrid baselines in this work serve as partial validation of SCoNN’s structural advantages.

Although the reported results demonstrate strong accuracy across datasets, this study currently reports mean accuracy values without explicit statistical indicators such as variance, standard deviation, or confidence intervals. Future work will incorporate detailed statistical validation, including k-fold cross-validation and confidence interval estimation, to provide a more comprehensive assessment of SCoNN’s robustness and generalization capability.

### Comparative analysis

In this section, a detailed comparative analysis of the proposed approach with existing methods is conducted with a focus on accuracy. To ensure an unbiased and fair comparison, only approaches that have been evaluated on the same dataset and using the same validation criteria as our proposed method have been shortlisted. This careful selection process allows for a direct and equitable assessment of the performance of different models under identical conditions, eliminating any potential biases that might arise from variations in datasets or validation techniques.

#### Comparative analysis of the RAVDESS dataset

This analysis compares the proposed SCoNN architecture with those of various researchers using the RAVDESS dataset. The focus of this study includes seven emotion classes extracted from the dataset: angry, happy, sad, calm, disgustful, fearful, and surprised. The results are categorized into two main groups.

Table 14 provides a detailed overview of several models proposed by different authors over time. Each utilizes various features and methodologies for emotion recognition from audio signals. One key aspect to note is the diversity in the feature sets used across these models. For instance, Issa et al.^16^ combined Mel Spectrogram, Chromagram, Spectral Contrast, MFCC, and Tonnetz features with a 1-D CNN architecture, achieving an accuracy of 71.61%. Alnuaim et al.^20^ focused on MFCC, STFT, and Mel spectrogram features with a multilayer perceptron (MLP) approach and achieved an accuracy of 81.00%. Similarly, other models, such as those of Andayani et al.^21^ with MFCC features and an LSTM-Transformer model, Kakuba et al.^23^ with MFCC, Chromagram, and Mel Spectrogram features using ABMD, and Dolka et al.^14^ with MFCC features and an artificial neural network (ANN), demonstrate varying levels of accuracy ranging from 75.62% to 88.72%. In contrast, the proposed SCoNN model introduces a novel approach combining the Mel Spectrogram and MFCC features. This combination seems promising because it captures spectral and temporal information crucial for emotion recognition. The methodology behind SCoNN, likely involving a specialized Spectrogram Convolutional Neural Network architecture, showcases a significant increase in accuracy, achieving an impressive 93.30%. This substantial improvement in accuracy compared to traditional CNNs, MLPs, LSTMs, and other architectures highlights the effectiveness of the proposed approach.

**Table 14:** Comparison of the accuracy of the proposed sconn model with that of existing approaches for the RAVDESS dataset.

Authors/Year	Features Used	Methodology	Accuracy
Issa et al.^16^	Mel Spectrogram, Chromagram, Spectral Contrast, MFCC, Tonnetz,	1-D CNN	71.61%
Alnuaim et al.^20^	MFCC, STFT, Mel Spectrogram	MLP	81.00%
Andayani et al. ^21^	MFCC	LSTM-Transformer	75.62%
Kakuba et al.^23^	MFCC, Chromagram, Mel Spectrogram	ABMD	85.89%
Dolka et al.^14^	MFCC	ANN	88.72%
Jahangir et al.^24^	Spectral contrast, tonnetz, MFCCS, delta-MFCCS, delta-delta MFCCS	1-D CNN	90.60%
Li. et al.^40^	IMel Spectrogram, Mel Spectrogram	CNN-SSAE	83.18%
Bhattacharya et al.^41^	MFCC, Chroma, Tonnetz, Contrast, Mel Spectrogram	CNN	90.86%
Khan et al.^42^	-	DeepESN, Dilated CNN, Multi-Headed Attention Mechanism	77.02%
Proposed Work	Mel Spectrogram	SCoNN	90.63%
	MFCC		91.51%
	Mel Spectrogram + MFCC		93.30%

Moreover, compared with state-of-the-art models such as the CNN-SSAE (83.18%) and CNNs with various features (up to 90.86%), the SCoNN demonstrates substantial improvement, indicating its superiority in capturing nuanced emotional cues from audio data. This could be attributed to its ability to learn intricate patterns and dependencies between the Mel Spectrogram and MFCC features, leveraging the strengths of both spectral and temporal representations.

From this analysis, we can clearly see that our proposed architecture performs better than all the mentioned methods. The model proposed by Bhattacharya et al.^41^ is the only work that is close to our work, with 90.86% accuracy.

#### Comparative analysis for the SAVEE dataset

The comparative investigation of the SCoNN model, in relation to previous techniques, on the SAVEE dataset provides valuable insights into the performance and methodology of different models in emotion recognition. This thorough assessment offers an exhaustive understanding of how different features and structures contribute to the precision and accuracy of emotion recognition from audio data.

Table 15 presents various approaches and features employed by different researchers. Kakuba et al.^23^ achieved a significant accuracy of 93.75% by combining MFCC, Chromagram, and Mel Spectrogram features using an adaptive boosting and multimodal decomposition (ABMD) technique. Incorporating both spectral and temporal data improves the model’s capacity to detect complex audio patterns associated with emotions. Similarly, Jahangir et al.^24^ utilized a wide range of features, such as spectral contrast, Tonnetz, MFCCs, delta-MFCCs, delta-delta MFCCs, and a chromagram, in conjunction with a 1D CNN architecture, resulting in an accuracy of 93.75%. The vast range of features and the capacity of the 1-D CNN to interpret sequential input are key factors in its exceptional performance.

**Table 15:** Comparison of the accuracy of the proposed sconn model with that of existing approaches for the SAVEE dataset.

Authors/Year	Features Used	Methodology	Accuracy
Kakuba et al.^23^	MFCC, Chromagram, Mel Spectrogram	ABMD	93.75%
Dolka et al.^14^	MFCC	ANN	86.80%
Li. et al.^40^	Mel Spectrogram, Imel Spectrogram	CNN-SSAE	88.96%
Jahangir et al.^24^	Spectral contrast, tonnetz, MFCCS, delta-MFCCS, delta-delta MFCCS, and chromagram	1-D CNN	93.75%
Singh et al.^43^	MFCC, pitch, ZCR, RMS	SVM	77.38%
Mishra et al.^44^	MRVMMFCC, MRVMAE, MRVMPE	DNN	83.40%
Mountzouris et al. ^45^	MFCC	CNN + ATN	74.00%
Saeed et al. ^46^	MFCC, Mel Spectrogram, Chroma, Poly Feature	DNN	90.00%
Liu et al.^47^	MFCC, Chromarequency, ZCR, MFCC, Chroma, Mel Spectrogram, Spectral Centroid, Spectral Contrast	CNN-A-LSTM	94.50%
Li et al. ^48^	Log Mel Spectrogram	DeepCNN	92.97%
Proposed Work	Mel Spectrogram	SCoNN	94.76%
	MFCC		91.43%
	Combined		95.00%

Conversely, certain methods show only average performance because of restrictions in choosing features or the methodology used. For example, Singh et al.^43^ employed Mel-frequency cepstral coefficient (MFCC), pitch, zero crossing rate (ZCR), and root mean square (RMS) features in conjunction with a support vector machine (SVM), achieving an accuracy rate of 77.38%. When each variable has its value, the SVM may not effectively utilize the intricate connections between them, resulting in inferior performance compared to models built on neural networks. In a similar manner, Mountzouris et al.^45^ employed Mel-frequency cepstral coefficients (MFCCs) as features in a convolutional neural network (CNN) model enhanced with an attention (ATN) mechanism, resulting in an accuracy of 74.00%. Although attention processes can improve model performance by directing focus toward crucial aspects of the input, relying solely on the combination of MFCC may not be adequate for capturing all subtle emotional nuances.

The proposed SCoNN model is efficient because of its novel architecture and efficient feature integration. The model attains a precision of 94.76% when employing Mel Spectrogram characteristics and 91.43% when utilizing MFCC characteristics. Nevertheless, by integrating both the Mel Spectrogram and MFCC characteristics, the model achieved a peak accuracy of 95.00%. This notable enhancement emphasizes the synergistic relationship between both characteristics, with the Mel Spectrogram offering intricate spectral data and the MFCC capturing crucial temporal dynamics. The SCoNN architecture is expected to utilize convolutional layers to extract spatial characteristics from spectrograms. It may also integrate recurrent layers to capture temporal dependencies, strongly representing emotional states. Furthermore, the model’s effectiveness is highlighted compared to that of advanced approaches such as the CNN-SSAE, DeepESN, and CNN-A-LSTM. Liu et al.^47^ achieved an impressive accuracy of 94.50% by utilizing a wide range of features and implementing a CNN-A-LSTM model. This successful outcome highlights the advantages of integrating convolutional and recurrent neural networks. Nevertheless, the SCoNN model outperforms this model with 95.00% accuracy, suggesting that the architecture decisions and feature integration in SCoNN are better at capturing the subtle details in emotional audio data.

The exceptional performance of the SCoNN model on the SAVEE dataset can be attributed to its deliberate feature selection and inventive architectural design. The model utilizes a combination of Mel spectrograms and MFCC features to efficiently harness spectral and temporal information advantages. This results in a more precise and resilient emotion recognition system. This thorough assessment not only showcases the cutting-edge performance of the model but also offers significant insights into the importance of selecting appropriate features and architecture when creating efficient emotion identification systems from audio inputs.

#### Comparative analysis for the TESS dataset

The analysis of Table 16, which contrasts the accuracy of multiple models, including the proposed SCoNN, on the TESS dataset reveals significant observations about the efficiency of diverse techniques and feature sets in emotion recognition tasks.

**Table 16:** Accuracy of the proposed sconn model compared with those of other models on the TESS dataset.

Author’s Name	Features	Methodology	Accuracy
Dolka et al.^14^	MFCCS	Artificial Neural Network	99.52%
Tellai et al. ^25^	Mel Spectrogram, Mel Frequency Cepstral Coefficients	CNN-Transformer	99.42%
Choudhary et al.^49^	MFCC	CNN	97.10%
Huang et al. ^50^	MFCC	2-D CNN	85.00%
This Work	Mel Spectrogram	SCoNN	99.68%
	MFCC		99.93%
	Combined		99.93%

Table 16 outlines the performance of various techniques employing a variety of features and methodologies. Dolka et al.^14^ utilized Mel-frequency cepstral coefficients (MFCCs) as features in combination with an artificial neural network (ANN) to obtain an impressive accuracy of 99.52%. MFCCs are renowned for catching fundamental speech signal characteristics, and their utilization with an ANN, which excels at acquiring intricate patterns, leads to exceptional accuracy. Similarly, Tellai et al.^25^ combined a Mel Spectrogram and Mel Frequency Cepstral Coefficients (MFCC) in a CNN-Transformer framework, achieving a remarkable accuracy of 99.42%. This combination utilizes spectral and cepstral properties, and incorporating a transformer architecture improves the model’s capacity to grasp temporal relationships and distant connections in the data. Choudhary et al.^49^ employed MFCC features in conjunction with a convolutional neural network (CNN) to achieve an accuracy of 97.10%, which is impressive. The CNN successfully captures spatial hierarchies from the MFCC features. Nevertheless, it may not achieve the highest accuracy since it may not handle temporal dynamics as effectively as models incorporating transformer structures. Huang et al.^50^ achieved an accuracy of 85.00% by utilizing MFCC features with a 2-D CNN. The model’s significantly lower accuracy may be attributed to its limited capacity to properly utilize the temporal components of the speech signal, which are essential for emotion recognition. The model described in this study utilizes an SCoNN architecture, resulting in higher accuracy rates. The SCoNN achieved an accuracy of 99.68% while employing only Mel Spectrogram characteristics, demonstrating its ability to effectively capture complicated patterns and comprehensive spectrum information inside the spectrograms. The model demonstrated an exceptional accuracy of 99.93% when utilizing MFCC characteristics. Furthermore, when the Mel Spectrogram and MFCC data are combined, the model maintains this high level of accuracy. The consistent performance of the SCoNN architecture demonstrates its ability to properly handle both independent and combined feature sets, demonstrating its robustness.

The SCoNN architecture excels in capturing features at several scales because of its fractal design, which involves the repeated use of convolutional layers. This design facilitates the model’s ability to acquire local and global patterns more efficiently than conventional CNNs. The remarkable precision attained through MFCC features indicates that the SCoNN is proficient in extracting crucial temporal and frequency-based attributes necessary for emotion recognition. The model’s potential to integrate multiple types of information, as demonstrated by its ability to sustain peak accuracy when integrating Mel Spectrogram and MFCC data, leads to a more thorough understanding of the emotional content of speech. The SCoNN model also demonstrated exceptional performance on the TESS dataset, surpassing other cutting-edge models by a substantial margin. The exceptional precision of the system, particularly when utilizing a combination of features, unequivocally highlights the necessity of incorporating both spectral and temporal information for the identification of emotions. The SCoNN architecture, renowned for its capacity to extract features at multiple scales, has unequivocally proven to be highly effective in capturing the nuanced aspects of emotional speech. This accomplishment firmly establishes a new standard in the field, underscoring the crucial importance of advanced architectures and rich feature sets in the successful development of high-performance emotion identification systems. The improved accuracy of SCoNN confirms that its hierarchical stacking and adaptive feature fusion design contributes to better emotion-specific discrimination compared to baseline CNN models.

## Conclusion

This study investigated the performance of four different simple deep learning algorithms—CNN, LSTM, CNN + LSTM, and LSTM + RNN—and proposed a deep convolutional architecture named SCoNN. The SCoNN model has been tested on three publicly available datasets: the RAVDESS, TESS, and SAVEE datasets. We used 4 data augmentation techniques, noise injection, time stretching, time shifting, and pitch shifting, to increase the quantity and diversity of the dataset. We extracted various features from the audio files of the datasets, including the mel spectrogram, MFCC, chroma STFT, ZCR, RMS, and pitch frequency. Various preprocessing techniques have been used for audio signals before any feature extraction task begins, including techniques such as “silence removal”, “preemphasizing”, and “normalization”. The first experiment is conducted with only the four classifiers except SCoNN to identify the most important features required for further analysis. In the first experiment, the extracted features were input into four different deep learning models, i.e., CNN + LSTM, CNN, LSTM, and LSTM + RNN, and we achieved exceptional accuracies of 99.57, 99.75, 99.96, and 99.85, respectively, on the MFCC features of the TESS dataset; additionally, LSTM has evolved as the best deep learning model for detecting emotions from MFCC features. In the second experiment, the MFCC features of the audio files are fed into the SCoNN framework. The experiment was conducted individually for each dataset and each feature segment, where 99.93% accuracy on the TESS dataset was achieved. In summary, the SCoNN architecture proved to be state-of-the-art compared to the existing approaches.

## Data Availability

The datasets used for the experiment and analysis in this article are available publicly. Corresponding links to the datasets are: RAVDESS:https://www.kaggle.com/datasets/uwrfkaggler/ravdess-emotional-speech-audioSAVEE:https://www.kaggle.com/datasets/barelydedicated/savee-databaseTESS:https://www.kaggle.com/datasets/ejlok1/toronto-emotional-speech-set-tess.

## Declarations

### Use of LLM

This article extensively used Large Language Models (LLM) tools to improve English writing, grammar, and punctuation.
